# Microring Resonator Dispersion Metrology With Neural Networks

**DOI:** 10.1002/nap2.70269

**Published:** 2026-08-31

**Authors:** Ergun Simsek, Shao‐Chien Ou, Gregory Moille, Kartik Srinivasan

**Affiliations:** ^1^ University of Maryland Baltimore Maryland USA; ^2^ Microsystems and Nanotechnology Division National Institute of Standards and Technology Gaithersburg Maryland USA; ^3^ Joint Quantum Institute NIST/University of Maryland College Park Maryland USA

**Keywords:** dispersion metrology, integrated dispersion, inverse design, material dispersion, neural networks, ring resonator

## Abstract

Precise knowledge of resonator dispersion, from both geometric and material contributions, is essential for reliable high‐performance nonlinear integrated photonics devices, such as optical parametric oscillators, frequency doublers, and integrated optical frequency combs. However, direct measurements at the fabrication level provide limited knowledge, whether through destructive cross‐section imaging or nondestructive ellipsometry, whereas complete optical characterization that enables precise dispersion metrology is time‐consuming and poorly suited for mass‐scale foundry fabrication. In this work, we develop a machine learning framework to solve three complementary problems: (i) predicting resonator geometric dimensions, (ii) identifying the correct material dispersion, and last, but not least, (iii) precisely reconstructing the integrated dispersion spectrum directly from ring dimensions. These three neural networks together enable both inverse and forward characterization of microring resonators. Using numerically generated datasets based on Sellmeier‐type material models, we demonstrate <1 nm ring dimension prediction accuracy without noise, <8 nm prediction accuracy with ≈45 dispersion samples under a realistic frequency measurement noise level (±50 MHz), and ≈16 nm prediction accuracy at a higher noise level (±200 MHz). The Sellmeier model classification exceeds 99% accuracy in all cases. Importantly, dispersion sampled far from the pump resonances proves most informative, reducing full‐spectrum characterization requirements. The forward‐prediction network reconstructs dispersion spectra from the ring dimensions with high accuracy, providing a rapid alternative to numerical simulations and enabling quick assessment of the dispersion regime. Additionally, we present two experimental case studies: in the first, we demonstrate that machine learning can infer trapezoidal ring cross‐sectional geometry, including sidewall angle, from measured integrated dispersion data; in the second, we validate our approach against a publicly available dataset, where ML‐predicted width and height show close agreement with independently measured values despite an unconstrained ring radius. By combining both inverse and forward predictions, our results highlight the potential of machine learning applied to dispersion data as a rapid, nondestructive tool for wafer‐scale quality control and process monitoring in photonic foundries.

## Graphical abstract

We present a machine learning framework for inverse and forward characterization of microring resonators, inferring geometry and material dispersion from sparse spectral data. The approach achieves nanometer‐scale accuracy and > 99% material identification, while reconstructing full dispersion spectra. This enables fast, nondestructive, wafer‐scale monitoring and a practical alternative to computationally intensive simulations.

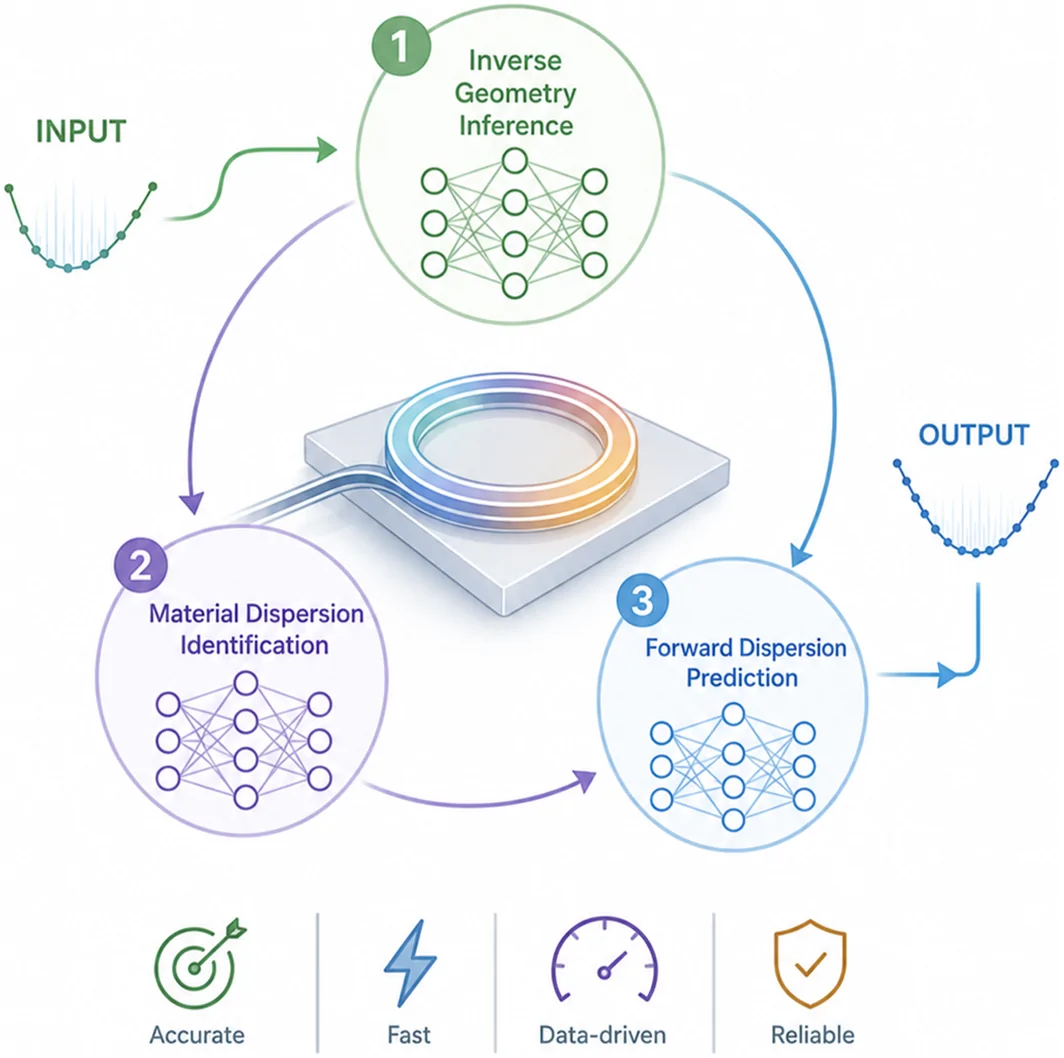

## 1 Introduction

The miniaturization and integration of optical frequency combs promises revolutionary advances in telecommunications [1], precision metrology [2], and quantum information science [3]. Among various approaches, optical frequency comb generation using the Kerr nonlinearity in microresonators (microcombs) has emerged as particularly promising due to its compact footprint, low power consumption, and potential for large‐scale integration [4, 5, 6, 7]. In particular, silicon nitride (${\text{Si}}_{3}N_{4}$) microring resonators have emerged as a platform of choice because of their low propagation loss, high Kerr nonlinearity, and compatibility with standard CMOS fabrication processes [8, 9, 10, 11]. These properties, together with broadband dispersion engineering capability [12], have led to the demonstration of octave‐spanning dissipative Kerr soliton (DKS) combs directly on a chip [13, 14, 15, 16, 17, 18] (Figure 1a). Such octave‐spanning DKS microcombs can be self‐referenced to stabilize their carrier‐envelope offset frequencies [19, 20, 21], opening the door for many applications in optical frequency synthesis, optical atomic clocks, ultra‐low noise microwave generation, and spectroscopy.

**FIGURE 1:**
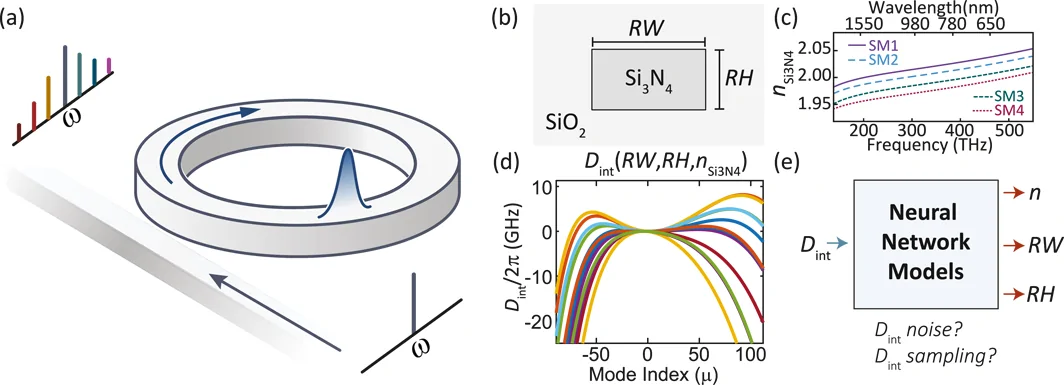
(a) Dissipative Kerr soliton (DKS) microcombs convert a single frequency laser to a frequency comb, often in ${\text{Si}}_{3}N_{4}$ microring resonators. The properties of the frequency comb depend on the dispersion of the microring resonator, quantified by the integrated dispersion $D_{\text{int}}$, which describes the deviation of the microring mode frequencies from a uniformly spaced grid. $D_{\text{int}}$ depends on (b) the resonator geometry, and in particular, the ring width (RW) and ring height (RH) and (c) the refractive index dispersion of the constituent materials. For ${\text{Si}}_{3}N_{4}$, its dispersion will be impacted by different gas precursor ratios in the growth process (labeled SM1 to SM4 here, and described in further detail later). (d) Example simulated $D_{\text{int}}$ curves for different geometric and material parameters. (e) Here, we use neural networks to understand how well we can extract geometric and material parameters given $D_{\text{int}}$ data. We further consider how well this extraction works in the presence of noise on the $D_{\text{int}}$ data associated with measurement uncertainty, and what level and types of downsampling of $D_{\text{int}}$ data can still lead to good parameter extraction.

For a usable octave‐spanning DKS microcomb, the resonator dispersion is typically tailored to enable phase matching between the DKS and the microring resonance. This phase matching generates dispersive waves [22], engineered to be at quasi‐harmonic frequencies [23], which enhance the power of phase‐matched comb teeth and drastically improve microcomb self‐referencing [19, 21]. The resonator dispersion has two contributions, a geometric component that is largely determined by the ring cross‐section (Figure 1b) and a material component that comprises both core and cladding contributions (Figure 1c). The fabrication precision directly impacts the geometric dispersion, as even ≲10 nm changes in ring width [15, 23] and thickness [24] can modify the comb bandwidth and shift dispersive wave frequencies by >5 THz. The material growth directly determines the material dispersion, which for amorphous materials such as Si_3_N_4_ is sensitive to the growth parameters such as the gas settings [25].

Methods for in‐line nondestructive monitoring remain limited to ellipsometry measurements on a restricted set of test wafers and exclude contributions from geometrical dispersion. To monitor this second contribution, destructive analysis is necessary to cross‐section a given set of fabricated microring resonators. Thus, such methods can be insufficient for optimization and monitoring of large‐scale fabrication processes, such as those at the American Institute for Manufacturing Integrated Photonics (AIM Photonics), where substantial enough variations can occur across a single wafer [26] to strongly impact microcomb performance [27]. An alternative, more direct, and nondestructive approach characterizes the microring resonator optically by measuring the residual dispersion of mode μ (Figure 1d), also called the integrated dispersion $\left(D_{\text{int}}\left(\mu \right)\right)$, against a fixed grid of frequency markers. However, complete characterization of a given resonator is time‐consuming, may require a large set of tunable lasers, and is therefore unsuitable for rapid testing of many fabricated devices for dispersion calibration across a wafer.

Recent work has highlighted the promise of machine learning (ML) and inverse‐design methods for addressing such topics. For example, ML‐based inverse design has been successfully applied to waveguide dispersion engineering, demonstrating agile dispersion profiles for broadband and nonlinear applications [28]. Similarly, random forest and decision tree regressors have been used to infer resonator geometries directly from dispersion measurements, with experimental verification on integrated ${\text{Si}}_{3}N_{4}$ platforms [29]. In ref. [30], Soroush et al. showed that broadband resonator‐waveguide coupling coefficients can be predicted from a small set of measured (or calculated) coupling coefficient values. More generally, an automated inverse design of on‐chip resonators has been demonstrated, overcoming long‐standing challenges associated with the highly nonconvex optimization landscape of resonant devices [31].

The growing adoption of ML in photonics extends well beyond these examples. In the context of quantum photonics, physics‐informed ML frameworks have been proposed to accelerate the discovery and integration of single‐photon sources, as well as to enable scalable assembly of quantum photonic circuits [32]. Complementary to ML‐driven design, advanced measurement and characterization techniques have also emerged, such as broadband interferometric methods for simultaneously extracting waveguide geometry and refractive indices with high precision, providing critical data for both model training and fabrication control [33]. In the area of microresonator‐based frequency combs, hybrid approaches combining deep neural networks with optimization algorithms have enabled accurate inverse design from target soliton microcomb spectra to cavity geometries, significantly reducing reliance on computationally intensive electromagnetic simulations [34]. Furthermore, ML techniques are increasingly being employed for real‐time analysis and control, including high‐accuracy classification of dissipative Kerr soliton states using hybrid deep learning architectures, enabling robust and automated operation of microcomb systems [35].

In this work (Figure 1e), we build upon these concepts and address three complementary problems using neural networks trained on numerically generated $D_{\text{int}}\left(\mu \right)$ datasets and ring dimensions. First, we demonstrate inverse prediction of microring geometry: given integrated dispersion measurements, our regression model infers the ring height and width with <1 nm accuracy in noiseless conditions. Second, we classify the material dispersion model by training a neural network to discriminate among different Sellmeier‐type refractive index models, achieving an accuracy exceeding 99%. This classification task complements ellipsometry: When a test wafer or witness sample is available, ellipsometry remains the most direct route to material dispersion. However, when such samples are absent, or when a rapid, in‐line consistency check is needed using data already being collected for geometric metrology, for example, to flag a deposition‐run drift across a wafer, the classifier provides a dispersion‐data‐only alternative that requires no additional measurement infrastructure. Third, we present a forward‐prediction neural network that estimates the coefficients of a sixth‐order polynomial to reconstruct the integrated dispersion spectrum directly from the ring dimensions. This forward model enables a rapid evaluation of $D_{\text{int}}$ without repeated numerical simulations and establishes a bidirectional ML framework linking geometry, material models, and spectral signatures. In other words, this forward model can be used as a predictive tool to determine whether a planned resonator design will yield a $D_{\text{int}}$ spectrum consistent with the desired dispersion regime. In this way, our framework not only enables robust geometry and material classification from spectral data but also provides a direct route to evaluating and optimizing dispersion profiles during the design stage. We validate the practical applicability of our approach through an experimental case study, demonstrating that the framework can recover trapezoidal cross‐sectional geometry from measured dispersion data, in the possible presence of fabrication‐induced deviations from ideal structures.

We further investigate the robustness of our models under realistic measurement noise and analyze the trade‐off between accuracy and the number of $D_{\text{int}}$ samples used, with particular emphasis on the practical limitations of dispersion measurements. In many experimental settings—especially in octave‐spanning microcomb studies—the accessible spectral range is constrained by the finite tuning bandwidth of individual lasers, often requiring multiple tunable sources to cover the full spectrum. As a result, acquiring densely sampled $D_{\text{int}}$ data across a wide bandwidth can be time‐consuming and experimentally demanding, effectively creating a bottleneck in device characterization. In this context, our approach leverages machine learning to extract maximum information from a limited number of strategically selected data points. Our results highlight two key findings: (i) in the presence of realistic measurement uncertainty of individual resonance frequencies of approximately ±50 MHz, < 8 nm accuracy can be obtained using as few as 45 $D_{\text{int}}$ samples, provided that the samples are chosen sufficiently far from the pump frequency, and (ii) for less precise systems with noise levels on the order of ±200 MHz, the expected errors approximately double, reaching ≈16 nm. These results directly address practical experimental constraints by quantifying the number of dispersion samples required to achieve a target accuracy, thereby enabling more efficient measurement strategies when full spectral coverage is not readily available. In this way, our framework reduces reliance on dense spectral scans and mitigates the need for multiple wideband tunable sources, offering a pathway to faster, more resource‐efficient characterization of integrated photonic resonators.

## 2 Integrated Dispersion Calculations

We consider a microring resonator composed of ${\text{Si}}_{3}N_{4}$ as the core material and ${\text{SiO}}_{2}$ as the cladding. The outer ring radius is fixed at 23μm, whereas the ring height and width are varied to construct a comprehensive dataset for dispersion analysis. Specifically, the height is swept from 620 to 720 nm and the width is swept from 750 to 950 nm in increments of 10 nm, resulting in 441 unique training designs. Additionally, we generate a test dataset by randomly selecting 50 distinct integer width–height combinations (in nanometers) from the same ranges as the training dataset.

To accurately capture material dispersion, we employ wavelength‐dependent relative permittivity models for both ${\text{Si}}_{3}N_{4}$ and ${\text{SiO}}_{2}$. The relative permittivity of ${\text{Si}}_{3}N_{4}$ under different gas ratios is modeled as

(1) $$\varepsilon_{r}^{{\text{Si}}_{3}N_{4}}\left(\lambda \right)=1+\frac{A}{1-{\left(B/\lambda \right)}^{2}}-C\lambda^{2},$$

where λ is the wavelength in μm and the parameters (A,B,C) depend on the gas ratio used during deposition [25]. The parameter sets are summarized in Table 1.

**TABLE 1:** Dispersion model parameters for $\varepsilon_{r}$ of ${\text{Si}}_{3}N_{4}$ at different gas ratios [25].

Model name	Gas ratio (${\text{NH}}_{3}$:${SiH}_{2}{\text{Cl}}_{2}$)	A	B	C
${SM}_{1}$	3:1	3.025	0.13534	0.0230
${SM}_{2}$	5:1	2.973	0.13475	0.0220
${SM}_{3}$	7:1	2.883	0.13364	0.0244
${SM}_{4}$	15:1	2.842	0.14018	0.0181

For the surrounding ${\text{SiO}}_{2}$, we adopt the Sellmeier‐type relation [36].

(2) $$\varepsilon_{r}^{{\text{SiO}}_{2}}\left(\lambda \right)=1+\sum_{i=1}^{3}\frac{A_{i}}{1-{\left(\frac{B_{i}}{\lambda }\right)}^{2}}.$$

The coefficients are given in Table 2.

**TABLE 2:** Dispersion model parameters for ${\text{SiO}}_{2}$.

*i*	$A_{i}$	$B_{i}$
1	0.6961663	0.0684043
2	0.4079426	0.1162414
3	0.8974794	9.896161

With these permittivity models, the calculation of $D_{\text{int}}$ proceeds as follows. We first compute the effective modal indices, $n_{\text{eff}}\left(\lambda \right)$, using our eigenmode solver [11, 37] at 171 discrete wavelength values uniformly distributed between 750 nm and 1.6μm for the training and test datasets. The propagation constant is given by $\beta \left(\lambda \right)=2\pi n_{\text{eff}}\left(\lambda \right)/\lambda$, and the azimuthal mode number m satisfies the resonance condition $m=\beta R_{c}$, where $R_{c}$ is the central radius of the microring. Since the values obtained directly from $\beta R_{c}$ are generally noninteger, we first fit a fifth‐order polynomial to the $n_{\text{eff}}$ versus m relationship and then evaluate the effective index at integer mode numbers $m_{i}$ within the physically relevant range. The resonance frequencies are then computed as follows:

(3) $$f_{i}=\frac{cm_{i}}{n_{\text{eff},i}L},$$

where $L=2\pi R_{c}$ is the ring perimeter and c is the speed of light.

We define the pump mode as the resonance closest to the target pump wavelength of 1060 nm. The free spectral range $D_{1}$ at the pump frequency can be calculated from $D_{1}=f_{1}-f_{0}$ or from $D_{1}=\left(f_{1}-f_{-1}\right)/2$, where $f_{0}$ is the pump resonance frequency and $f_{-1}$ and $f_{1}$ are the resonance frequency of the adjacent modes. Then, the integrated dispersion is evaluated as a function of relative mode number $\mu =m_{i}-m_{0}$, where $m_{0}$ is the pump mode number:

(4) $$D_{\text{int}}\left(\mu \right)=f_{\mu }-\left(f_{0}+D_{1}\mu \right),$$

where $f_{\mu }$ is the resonance frequency corresponding to relative mode number μ. This procedure yields 201 integrated dispersion values at integer relative mode numbers, determined from the original 171 wavelength points. Although our primary analysis focuses on these $D_{\text{int}}$ profiles, we later incorporate $D_{1}$ as a supplementary feature to account for its influence on ring dimension predictions and to evaluate the model's robustness against frequency offsets.

Figure 2 shows the integrated dispersion curves as a function of mode number for different precursor gas ratios of 3:1 (${\text{SM}}_{1}$), 5:1 (${\text{SM}}_{2}$), 7:1 (${\text{SM}}_{3}$), and 15:1 (${\text{SM}}_{4}$). At low gas ratios, the dispersion curves spread out more significantly, especially at large negative mode indices. This means that changes in geometry (ring width/height) lead to large and distinguishable changes in $D_{\text{int}}$. With increasing gas ratios, the $D_{\text{int}}$ curves become much more compressed. In this regime, the sensitivity of the integrated dispersion to geometric variations is reduced; that is, different ring dimensions yield increasingly similar $D_{\text{int}}$ profiles. This reduced parametric sensitivity implies that the input features carry less unique information about the underlying geometry, making the inverse mapping more susceptible to noise. Consequently, although the neural network can still perform reliably on noiseless synthetic datasets where subtle differences remain discernible, its predictive accuracy is expected to degrade in experimental conditions where random noise is unavoidable.

**FIGURE 2:**
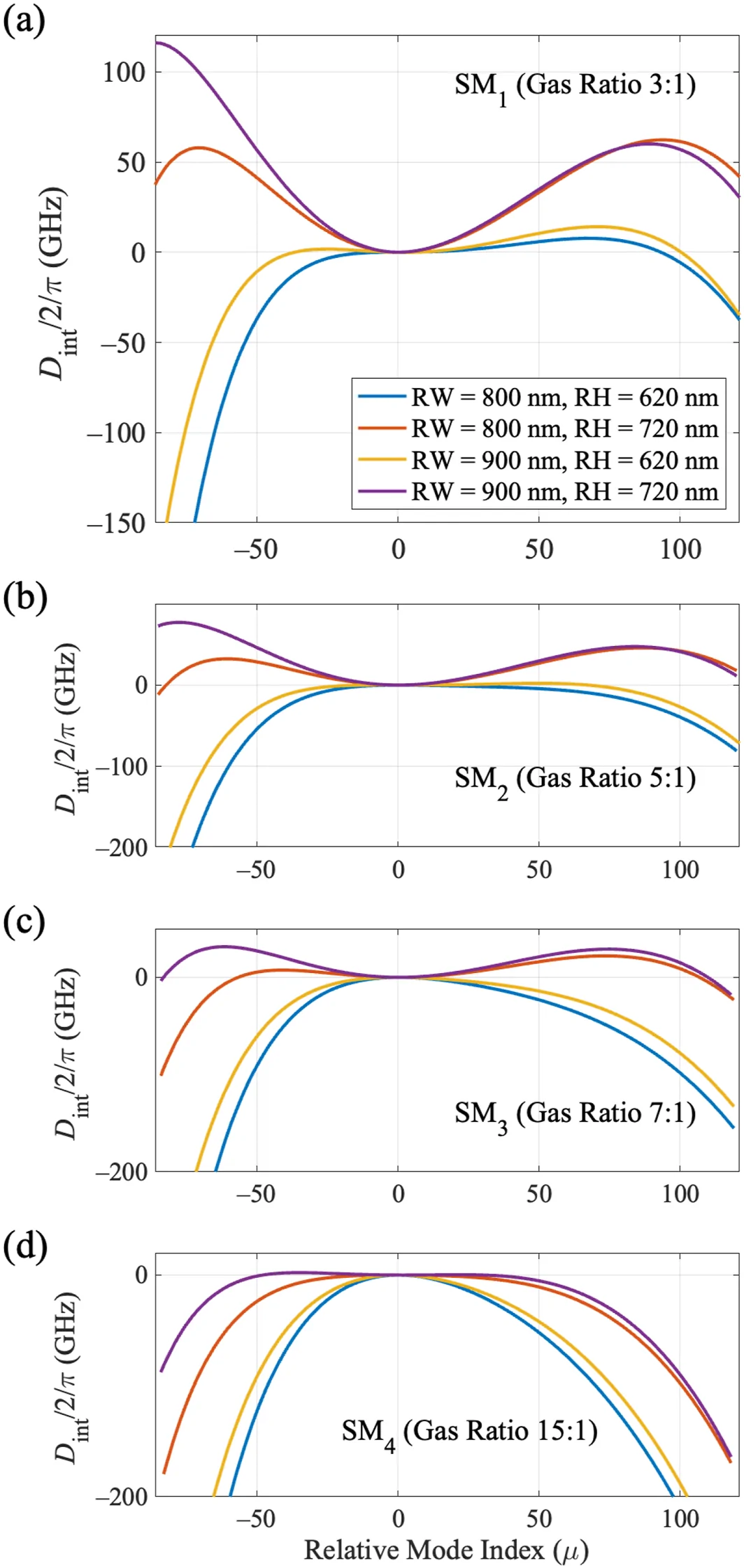
Integrated dispersion curves $\left(D_{\text{int}}/2\pi \right)$ as a function of mode index for ${\text{Si}}_{3}N_{4}$ films grown with the four precursor gas ratios outlined in Table 1: (a) 3:1, (b) 5:1, (c) 7:1, and (d) 15:1. Each sub‐panel contains 4 distinct $D_{\text{int}}/2\pi$ curves for the ring width and height values mentioned in the legends of the top panel.

## 3 Numerical Results

In this study, we used three distinct neural network architectures to address three problems associated with the integrated dispersion curves of dielectric ring resonators, as illustrated in Figure 3.

**FIGURE 3:**
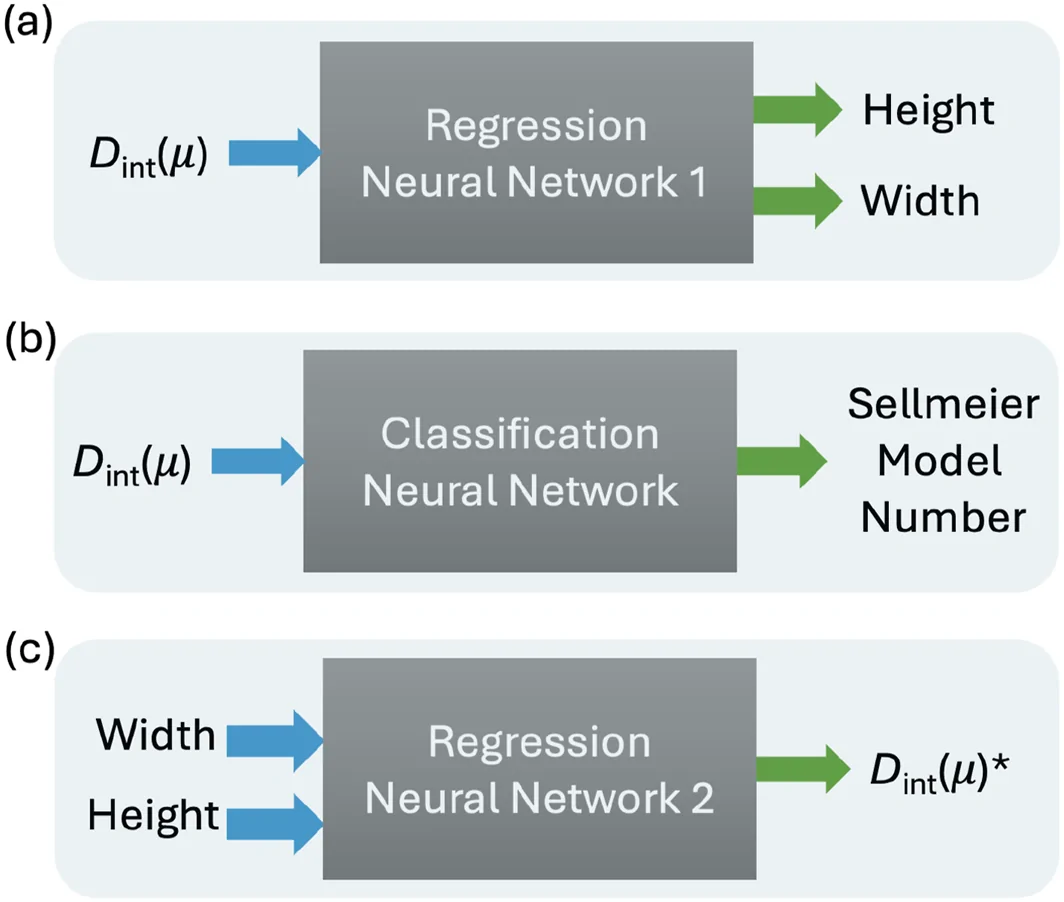
Schematic diagrams of the three neural network architectures used in this study: (a) Regression model for predicting the ring width and height from the integrated dispersion $\left(D_{\text{int}}\left(\mu \right)\right)$. (b) Classification model for identifying the Sellmeier model from dispersion data. (c) Another regression model for predicting the coefficients of a $6^{\text{th}}$ order polynomial fitting the integrated dispersions from the ring widths and heights. To emphasize that we regress on the polynomial coefficients, not on the integrated dispersion directly, we use an asterisk.

### 3.1 Dimension Prediction

The first network, Figure 3a, is designed for regression and aims to predict the width and height of the resonator from the $D_{\text{int}}$ data. In this setting, the input consists of the normalized $D_{\text{int}}$ values determined over 201 mode numbers, whereas the output is the width and height of the rings. The network consists of 20 fully connected hidden layers, each containing 64 neurons, with hyperbolic tangent (tanh) activation functions applied to introduce nonlinearity. The output layer contains two linear neurons corresponding to the width and height predictions. Training is performed using the Levenberg–Marquardt optimization algorithm [38] with a mean squared error loss function, a batch size of 32, and convergence typically achieved within 200 epochs. To evaluate the accuracy of the regression network in predicting the width and height of the ring resonators, we first train the model and then test it using datasets created with the Sellmeier model 1 (gas ratio of 3:1). The results are summarized in Figure 4 as follows.

**FIGURE 4:**
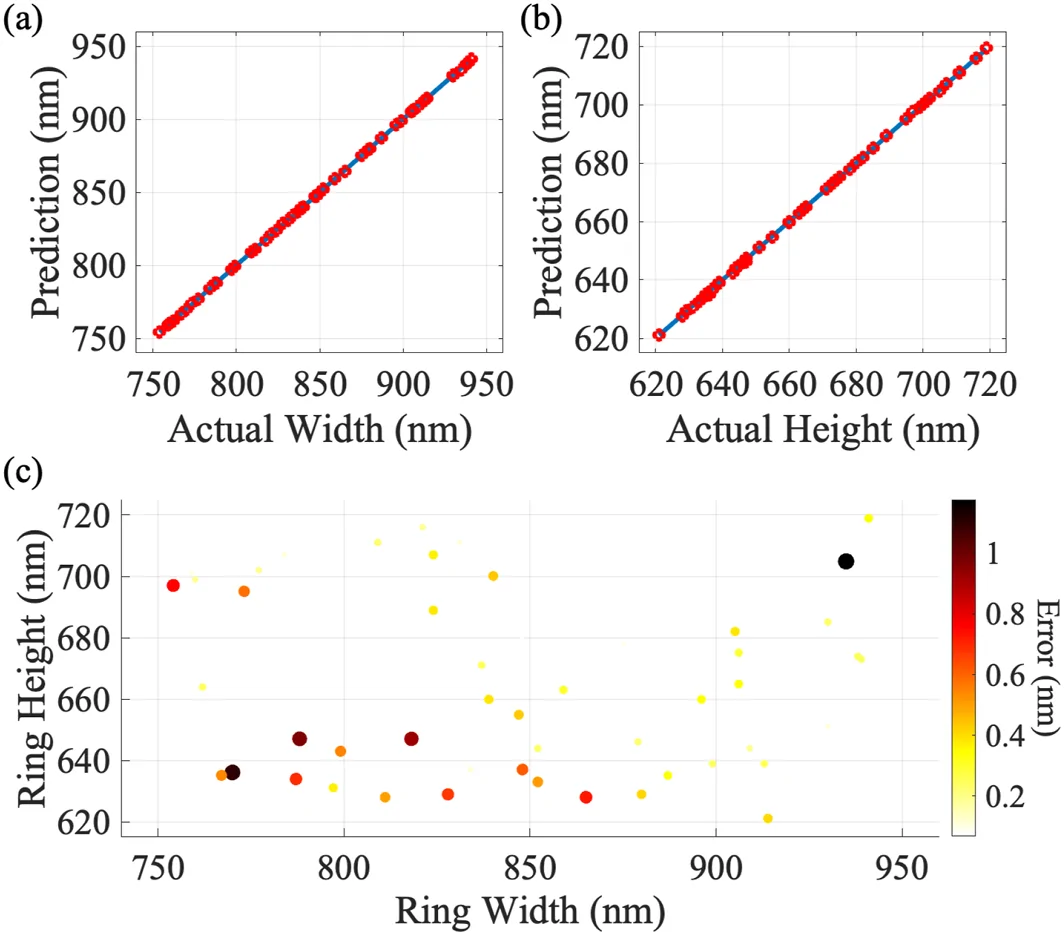
Performance of the regression neural network trained and tested on datasets generated using the Sellmeier model 1. (a) and (b) Predicted versus true values for the ring width and ring height, respectively. (c) Error distribution in the actual width–height plane. We calculate the overall error with the formula $\sqrt{{\left(w_{a}-w_{p}\right)}^{2}+{\left(h_{a}-h_{p}\right)}^{2}}$, where w and h represent width and height, and subscripts a and p refer to actual and predicted values.

The panels (a) and (b) of Figure 4 display the one‐to‐one correspondence between predictions and ground truth for ring width and height, respectively. In both cases, the data points are tightly clustered along the diagonal, indicating that the network is able to capture the mapping with remarkable precision. The near‐perfect linearity of these plots suggests that the model is not only accurate but also unbiased across the full parameter range of the test set. In panel (c), we plot the error over the actual width–height plane. The circles' radii increase, and their colors get darker, with increasing error. Although the vast majority of points exhibit excellent agreement (0.5 nm error), slightly larger deviations occur where the ring height is small. In this regime, the differential change in dispersion $\left(\partial D_{\text{int}}/\partial h\right)$ is minimized. Because the neural network relies on the spectral curvature—governed by higher‐order dispersion terms—the reduced sensitivity of these terms to height variations makes the inverse problem less well‐conditioned.

Figure 5 provides histograms of the absolute errors $\left|h_{a}-h_{p}\right|$ and $\left|w_{a}-w_{p}\right|$ for (a) height and (b) width prediction, respectively. These distributions confirm the high accuracy of the network: for 49 out of 50 test cases, the prediction error in both width and height was below 1 nm. We obtained similar results with the datasets created with the other three Sellmeier models. We do not provide them here for the sake of brevity.

**FIGURE 5:**
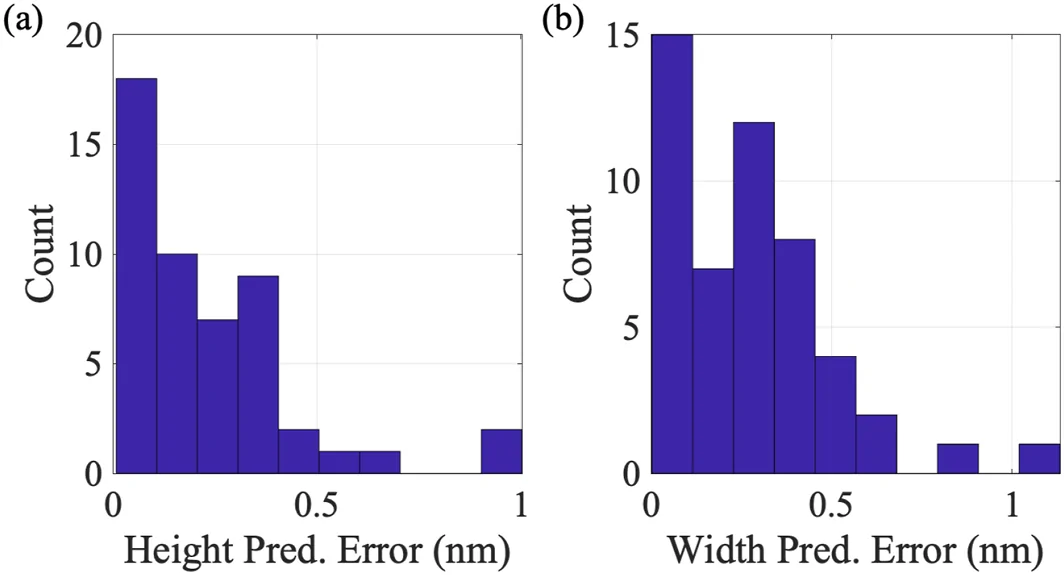
Histograms of the absolute prediction errors for (a) height and (b) width, respectively, for the regression neural network studied in Figure 4. In 49 out of 50 test cases, the errors remain below 1 nm.

To benchmark the performance of our regression network against alternative machine‐learning approaches, we also evaluated several standard models—including linear regression, decision trees, random forests, support vector machines, and Gaussian process regression—using the same training and test datasets. The results, summarized in Table 3, show that although the Gaussian process model achieves the lowest mean prediction error, its computational cost is nearly an order of magnitude higher than that of the neural network. Conversely, simpler models such as linear regression and decision trees train rapidly but exhibit larger errors, indicating that they lack the expressive capacity needed to capture the nonlinear relationship between integrated dispersion and ring dimensions. Random forests and support vector machines perform moderately well but still fall short of the neural network's accuracy. Overall, the neural network offers the best balance between prediction accuracy and training time, delivering <1 nm precision with manageable computational efforts. For this reason, we adopt neural networks as the primary modeling tool in the remainder of the manuscript.

**TABLE 3:** Mean width and height prediction errors and training times of six machine learning models.

ML model	Mean width Pred. error (nm)	Mean height Pred. error (nm)	Training time (s)
Linear regression	1.2516	1.7294	0.01
Decision trees	8.7568	2.3719	0.05
Support vector	3.0871	1.5593	18.7
Random forest	5.1966	1.3019	19.3
Neural network	0.2778	0.2323	84.1
Gaussian process	0.0663	0.0526	277

In practical scenarios, experimental measurements are inevitably subject to uncertainties introduced by the finite precision of the measurement system. To emulate such conditions, we introduce controlled noise into both the training and testing datasets. Specifically, the integrated dispersion values are perturbed according to the following formula:

(5) $$D_{\text{int}}^{\text{noisy}}=D_{\text{int}}+2\times \left(\text{rand}\left(m,\mu \right)-0.5\right)\times \Delta f,$$

where rand(m,μ) generates uniformly distributed random values in the interval [0, 1], and Δf denotes the noise level and m is the number of designs. To represent a high precision measurement system, we set Δf=50MHz (consistent with the performance of many commercial laser wavemeters). Conversely, to emulate a lower‐precision measurement system, we set Δf=200MHz. These choices allow us to assess the robustness of the regression network at different levels of measurement uncertainty.

Table 4 reports the prediction errors for the ring width in different gas ratios and noise conditions. In the absence of noise, the neural network achieves <1 nm mean absolute errors (MAE) for all gas ratios, with maximum errors of approximately 1 nm, and at most one test case exceeding 10 nm, demonstrating high prediction accuracy. At moderate noise levels (±50 MHz), the average error increases slightly to a range of 0.7–2.1 nm, depending on the gas ratio. Still, the coefficient of determination $\left(R^{2}\right)$ remains above 0.99 for all gas ratios, indicating that the model can tolerate realistic perturbations in the data. For all gas ratios, there are a few cases (< 3) with errors higher than 10 nm. For the ±200 MHz random noise case, larger errors are observed, with MAEs reaching up to 8.5 nm and several test cases exceeding 10 nm, especially for high gas ratios. Nevertheless, $R^{2}$ values remain above 0.95, confirming that the model retains high predictive fidelity under stronger noise. Finally, we note that the MxAEs (the maximum absolute errors of all the test cases) are much larger for the ±200 MHz random noise case, particularly for the highest gas ratios.

**TABLE 4:** Width prediction errors for the neural network ML model.

Gas ratio	Noise level	MAE (nm)	MxAE (nm)	$R^{2}$	$\#\left\{\Delta \right.$ > $\left.10\;\text{nm}\right\}$
3:1	No noise	0.278	1.133	0.99996	1
	50 MHz	1.336	35.469	0.9917	1
	200 MHz	2.712	17.027	0.9947	1
5:1	No noise	0.172	0.824	0.99998	0
	50 MHz	0.759	10.669	0.9989	1
	200 MHz	5.299	18.052	0.9850	6
7:1	No noise	0.259	1.049	0.99996	1
	50 MHz	2.104	16.20	0.995	2
	200 MHz	6.885	25.560	0.9744	12
15:1	No noise	0.264	1.1079	0.99996	1
	50 MHz	1.592	9.9694	0.9979	0
	200 MHz	8.507	45.525	0.9504	18

*Note:* #{Δ>10nm}: number of test cases where the error is larger than 10 nm.

Abbreviations: MAE, mean absolute error in nanometers; MxAE, maximum absolute error in nanometers of all the test cases.

Table 5 summarizes the ring height prediction errors. Without noise, MAEs remain well below 0.3 nm, with maximum errors near or below 1.5 nm and almost no cases exceeding 1 nm. Under ±50 MHz random noise, errors increase moderately (MAEs between 0.6 and 1.4 nm), but $R^{2}$ values stay above 0.986, confirming robust performance. For the ±200 MHz random noise case, deviations become more pronounced (MAEs up to 3.4 nm and maximum errors around 20 nm), with a few test cases exceeding 10 nm. Nevertheless, $R^{2}$ values remain above 0.96, indicating that the model continues to provide reliable predictions even in the presence of substantial noise. Overall, height prediction errors are consistently smaller than width prediction errors; this behavior can be understood from the fact that, within the geometries studied, $D_{\text{int}}$ is more sensitive to variations in height than in width. Consequently, the inverse mapping from dispersion to ring height is better conditioned, enabling the network to achieve higher height‐prediction accuracy.

**TABLE 5:** Height prediction errors for the neural network ML model.

Gas ratio	Noise level	MAE (nm)	MxAE (nm)	$R^{2}$	$\#\left\{\Delta \right.$ > $\left.10\;\text{nm}\right\}$
3:1	No noise	0.232	1.0018	0.99988	1
	50 MHz	0.614	13.073	0.9957	1
	200 MHz	1.037	10.111	0.9953	1
5:1	No noise	0.157	1.4685	0.99992	1
	50 MHz	0.587	7.348	0.9974	0
	200 MHz	2.535	15.471	0.9851	1
7:1	No noise	0.264	0.8533	0.99987	0
	50 MHz	1.419	16.105	0.9866	2
	200 MHz	3.171	15.397	0.9757	3
15:1	No noise	0.274	0.7385	0.99985	0
	50 MHz	1.351	6.760	0.9957	0
	200 MHz	3.399	20.465	0.9647	4

Note that the noise was added to the integrated dispersion data using a uniform (random) distribution rather than a Gaussian distribution. This choice represents a conservative and adversarial noise model, that is, the uniform noise assigns equal probability to all perturbation amplitudes within a fixed interval, thereby increasing the likelihood of large deviations compared to Gaussian noise with the same variance. If Gaussian noise were used instead, the resulting prediction errors in the dimension inference framework would be systematically smaller. This is because Gaussian noise is strongly concentrated around its mean (zero), with large‐amplitude perturbations occurring with exponentially decreasing probability. As a result, most dispersion samples would experience only small perturbations, preserving the smooth spectral structure and curvature of the integrated dispersion profile that are critical for accurate dimension extraction. In contrast, uniform noise introduces larger amplitude fluctuations that are uncorrelated across modes, leading to stronger distortion of higher‐order dispersion features. These features play a dominant role in constraining geometric parameters, and their degradation directly propagates into larger prediction errors.

To address the potential impact of experimental frequency shifts and fabrication‐induced deviations on the ring dimension prediction framework, we conducted an additional sensitivity analysis by introducing stochastic perturbations to the resonance frequencies. Recognizing that experimental jitter and thermal fluctuations can shift resonance positions, we simulated two distinct noise regimes by adding random frequency deviations of ±50 MHz and ±200 MHz to the calculated resonance frequencies in our test dataset. To assess the architecture's robustness, we first trained a neural network (a three‐layer deep regression neural network utilizing 256 neurons per hidden layer, leaky ReLU activation functions, and a 20% dropout rate for regularization, optimized via the Adam algorithm over 200 epochs) to predict the free spectral range, $D_{1}$, from the integrated dispersion dataset using our noise‐free training set (${\text{SM}}_{2}$). We then tested it on our noise‐added datasets. At a noise floor of 50 MHz, the model achieved an average root mean squared prediction error for $D_{1}$ of 0.079%. When we used our dimension‐prediction neural network on the integrated dispersion test dataset with slightly off $D_{1}$ values, we observed average errors of 0.18 nm for the ring width and 0.12 nm for the ring height. Our results demonstrate that the neural network exhibits high resilience to frequency noise, functioning as a nonlinear filter that prioritizes the global curvature of the dispersion profile over localized stochastic fluctuations. Remarkably, quadrupling the noise level to 200 MHz only marginally increased the $D_{1}$ prediction error to 0.088%, whereas the width and height average errors were determined to be 0.29 and 0.23 nm, respectively. The observed stability in $D_{1}$ prediction, despite a fourfold increase in frequency noise, can be attributed to the scale disparity between the optical frequencies and the MHz‐scale perturbations. Before proceeding to our next study, it is further worth noting that a neural network with only three hidden layers can predict $D_{1}$ from ring width and height with remarkable precision. Even with ±50 MHz of added noise, the average prediction error remains as low as 0.0139%. When the noise level is quadrupled to 200 MHz, the error rate increases only marginally to 0.0222%, demonstrating the model's high robustness.

For the initial study, we utilized the complete integrated dispersion dataset. To further investigate how many $D_{\text{int}}$ samples are required for accurate predictions, we designed a statistical sampling procedure. Specifically, we randomly select M number of $D_{\text{int}}$ samples from each of three regions: Region 1, Region 2, and Region 3, which include 67 modes from the left (mode indices from −91 to −25), middle (from −24 to 41), and right (from 42 to 109) portions of the spectrum, respectively (Figure 6). This results in a total of N=3M sampled data points. The random selection process was repeated 40 times to emulate a Monte Carlo–type analysis, allowing us to assess statistical variability. The parameter M was systematically varied from 2 to 16 in increments of 2, corresponding to total sample sizes N ranging from 6 to 48 in steps of 6. Two representative examples representing the lower and upper limits of this sampling are illustrated in Figure 6: (a) M=2 (N=6) and (b) M=16 (N=48).

**FIGURE 6:**
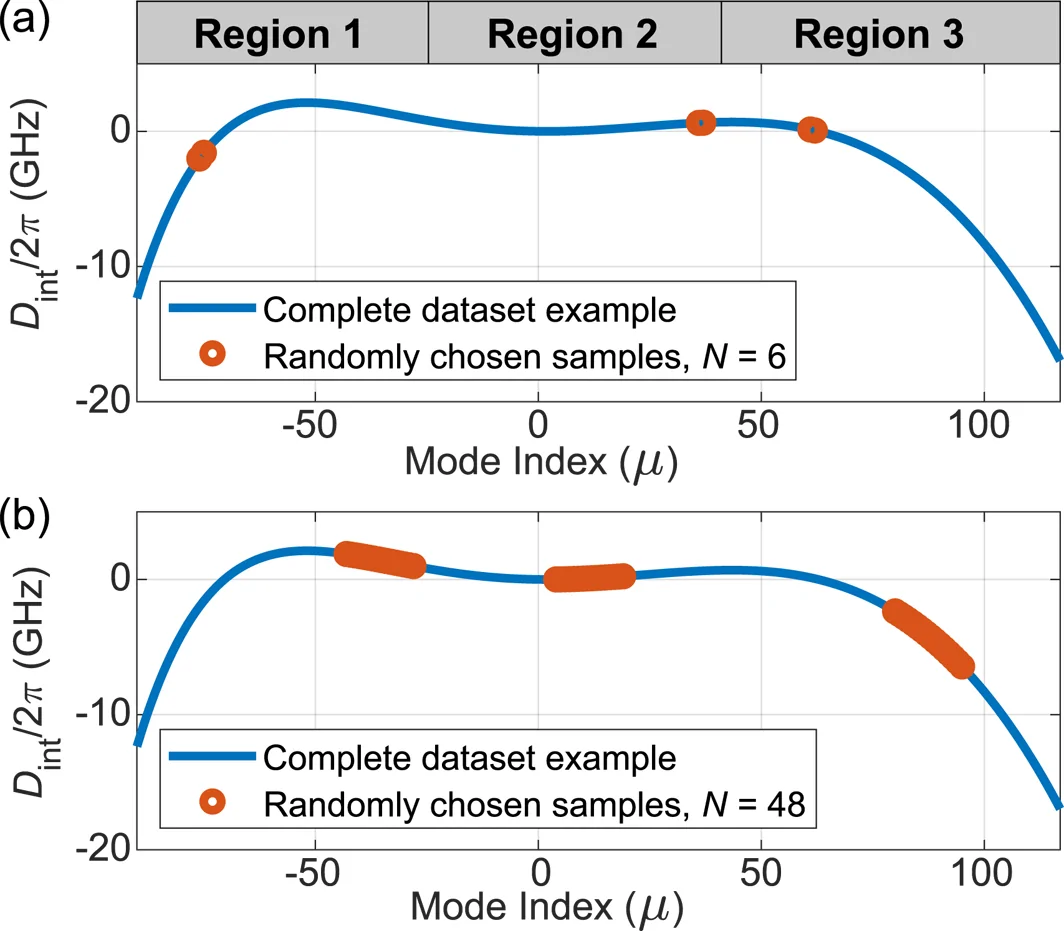
Examples of random sampling from the integrated dispersion dataset, where M random samples are chosen from each of Regions 1 (μ=−91 to μ=−25), 2 (μ=−24 to μ=41), and 3 (μ=42 to μ=109), for a total of N=3M samples. (a) Case for M=2 (N=6), and (b) case for M=16 (N=48). The blue line represents the complete $D_{\text{int}}/2\pi$ dataset, whereas the red circles denote M neighboring samples randomly chosen in three regions and used for training and testing.

The results in Figure 7 demonstrate that prediction accuracy strongly depends on the number of $D_{\text{int}}$ samples, noise level, and the gas ratio. Left column figures, panels (a), (c), and (e), report the average width prediction error, whereas (b), (d), and (f) in the right column show the height prediction error, with the rows corresponding to no noise, ±50 MHz noise, and ±200 MHz noise, respectively.

**FIGURE 7:**
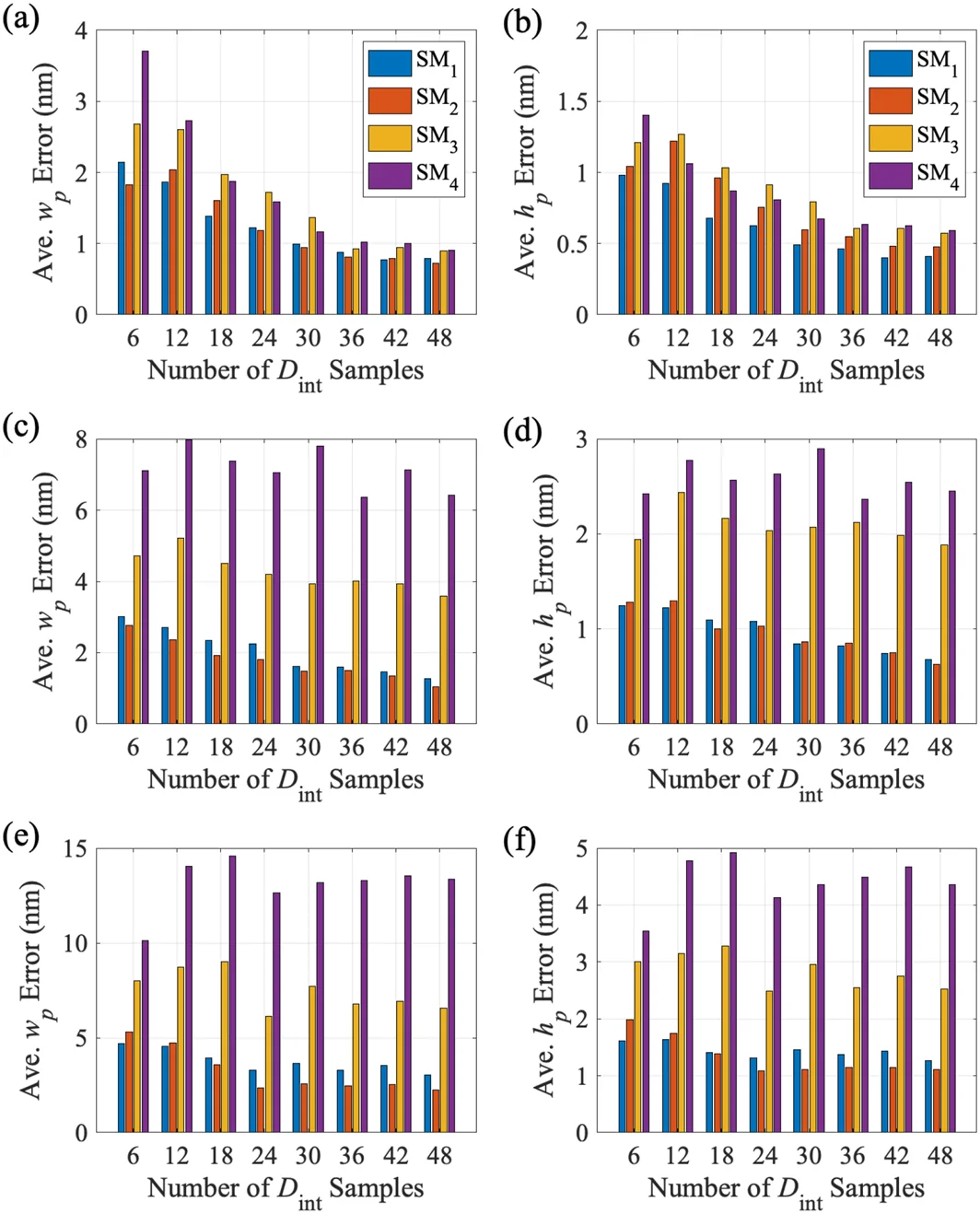
Left (right) column: average width (height) prediction error as a function of number of $D_{\text{int}}$ samples used in the neural network training and testing. The top, middle, and bottom rows are the cases with no noise, ±50 MHz random noise, and ±200 MHz random noise, respectively.

When there is no noise, using only six $D_{\text{int}}$ samples produces ring width and height errors that in all cases are <4 nm and <1.5 nm, respectively, with the errors reaching <1 nm with >40 $D_{\text{int}}$ samples. In all cases, a larger N improves accuracy, confirming that broader sampling reduces statistical variability. Once noise is added, the above trends hold for the ${\text{SM}}_{1}$ and ${\text{SM}}_{2}$ gas ratio cases, with the ring width and height errors ranging from <5 nm and <2 nm (N = 6), respectively, to <3 nm and <1.5 nm (N>40), respectively. However, for the noisy cases, we do not observe the same effect for the moderate and high gas ratios (${\text{SM}}_{3}$ and ${\text{SM}}_{4}$). As mentioned earlier, because the $D_{\text{int}}$ spectrum flattens with increasing gas ratios, the prediction errors also increase.

Going forward, the case N=45, that is, 15 $D_{\text{int}}$ samples recorded at wavelengths closer to the pump wavelength and two other ends of the spectrum, is especially important since it resembles our experimental setting (discussed later). Under realistic noisy conditions, N=45 yields width errors in the 2–8 nm range and height errors in the 1–3 nm for ±50 MHz random noise. For ±200 MHz random noise cases, the average width and height prediction errors are in the 4–14 nm and 1–5 nm, respectively. Overall, we can conclude that for the more precise measurement systems, 20 or more $D_{\text{int}}$ is sufficient to predict the ring dimensions with errors less than 8 nm, and having more $D_{\text{int}}$ samples helps with decreasing the prediction errors, especially if the ${\text{NH}}_{3}$‐to‐${\text{SiH}}_{4}$ gas ratio is kept low. For less precise measurement systems, one should expect higher errors. Moreover, as noted above, the performance with a significantly reduced number of samples (e.g., N=6) is still good for the ${\text{SM}}_{1}$ and ${\text{SM}}_{2}$ cases, with increased measurement throughput of potential practical interest.

We also investigate the impact of the spectral sampling location on the accuracy of width predictions using synthetic data generated from the ${\text{SM}}_{2}$ dataset with added 50 MHz random noise. To probe this effect, we constructed sub‐datasets by selecting either 25 or 45 consecutive $D_{\text{int}}$ values centered around a mode index $m_{c}$ (as illustrated in Figure 8a), repeating the process 1000 times for each $m_{c}$ to account for variability due to neural network initialization. Note that 25 and 45 consecutive $D_{\text{int}}$ values correspond to ≈10% and ≈20% of the entire octave‐spanning spectrum (750–1600 nm), respectively. The results in Figure 8b reveal a strong dependence on the sampling location when only 25 samples are used. In particular, if the sampling window is centered near the pump frequency, where $D_{\text{int}}$ values are small and nearly flat, the network receives little discriminative information and prediction errors rise sharply. By contrast, when the window is shifted away from the pump into regions where $D_{\text{int}}$ exhibits stronger curvature, the errors drop substantially, typically to the 8–15 nm range. Crucially, when the sampling window covers ≈20% of the spectrum (45 samples), every window inevitably includes some portion of sloped $D_{\text{int}}$. This demonstrates that reliable predictions require not only a sufficient number of samples but also that at least some of these samples be chosen away from the pump frequency, where the spectral features are more informative. That being said, we note that an implicit assumption of the above analysis is that the resonator $D_{1}$ coefficient (i.e., the free‐spectral range around the pump) is accurately known throughout.

**FIGURE 8:**
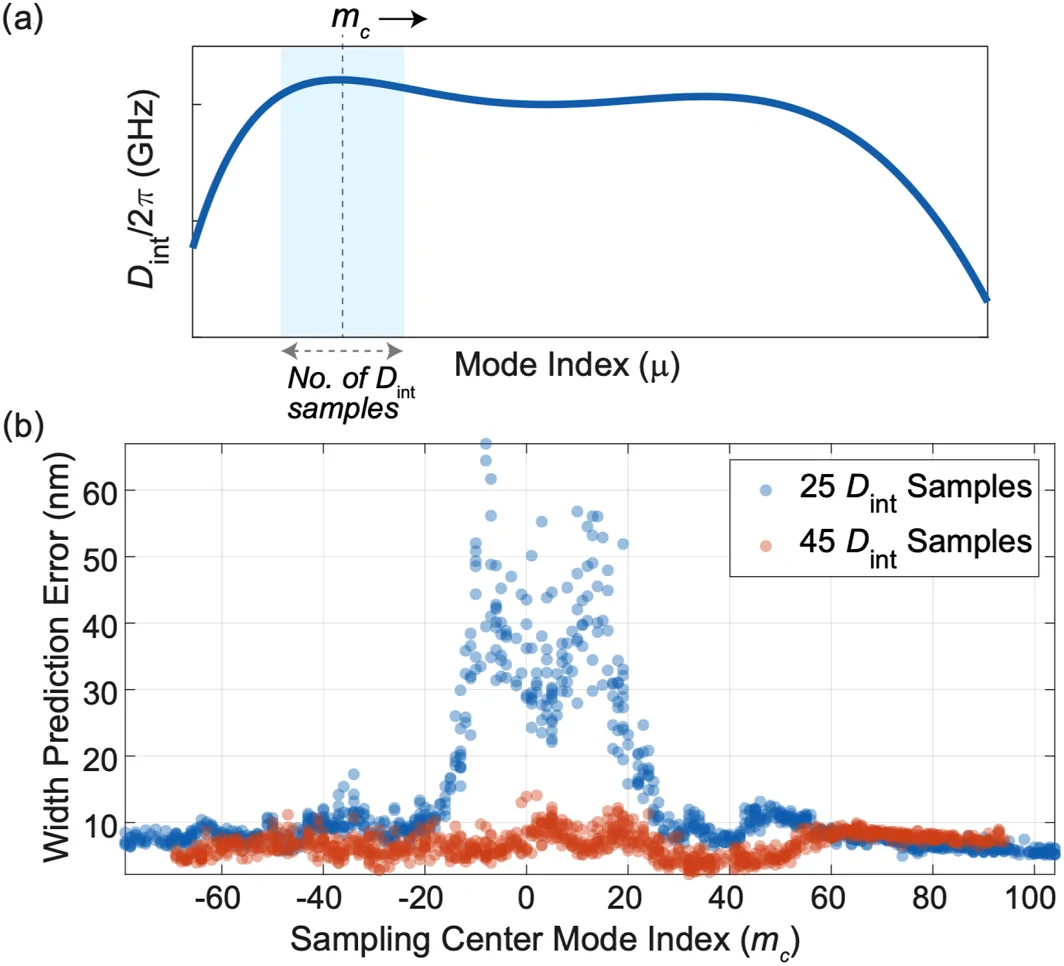
(a) Schematic for definitions of $m_{c}$ and the number of $D_{\text{int}}$ samples. (b) Width prediction error as a function of the sampling center mode number $m_{c}$, obtained from 1000 trials using the Sellmeier model‐1 dataset with 50 MHz random noise. For each trial, 25 (blue) and 45 (red) consecutive $D_{\text{int}}$ values centered at $m_{c}$ were used to train and test the neural network model. The resonator $D_{1}$ value is assumed to be well‐understood, independent of where the sampling takes place.

### 3.2 Sellmeier Model Classification

The second network, Figure 3b, handles a classification problem in which the goal is to identify the Sellmeier model used for the ${\text{Si}}_{3}N_{4}$’s relative electrical permittivity (refractive index) in $D_{\text{int}}$ calculations. Before describing the network architecture and results, we clarify the intended use case of this classifier and its relationship to the geometry‐prediction network of Section 3.1. In the current framework, the two networks are independent: the geometry‐prediction network assumes that the Sellmeier model is known a priori (e.g., from ellipsometry on a witness sample, as in our experimental case study), whereas the classification network operates as a separate tool that identifies the material model from $D_{\text{int}}$ data alone. Joint, simultaneous extraction of both material and geometric parameters is not performed in this work; we return to this point below. The classification network is therefore best suited to a specific and practical scenario: one in which a small, discrete set of candidate Sellmeier models is known a priori, for example, because a foundry uses a fixed menu of deposition recipes characterized ahead of time and the goal is to identify, from dispersion data alone, which recipe was actually realized for a particular device or wafer region. This is distinct from the open‐ended problem of inferring arbitrary Sellmeier coefficients from dispersion data. In this setting, the classifier provides a lightweight, dispersion‐data‐only consistency check, for example, flagging a deposition‐process drift between nominally identical runs using measurements already collected for geometric metrology and without requiring a separate ellipsometry step.

For this classification problem, the same normalized $D_{\text{int}}$ vectors serve as inputs, whereas the output is categorical, corresponding to one of four possible Sellmeier models (Table 1). The architecture comprises four hidden layers with progressively decreasing sizes of 128, 64, 32, and 16 neurons, respectively. The first two layers employed hyperbolic tangent (tanh) activations, whereas the subsequent two layers utilized rectified linear unit (ReLU) activations. A softmax layer with four neurons was used at the output to generate class probabilities. Training was conducted using the Adam optimizer with a categorical cross‐entropy loss function, a batch size of 64, and 100 epochs.

For the initial study, the training and testing inputs are 1764×201 and 200×201, respectively; the training and testing outputs are 1764×1 and 200×1, respectively. Figure 9 presents the confusion matrix that was obtained under all varying noise conditions. In all cases, the classifier achieves nearly perfect accuracy, with only a single misclassification observed between Classes 3 and 4 among 200 test cases. This result shows that, unlike the regression problem, where we predict the ring dimensions, the classification task of identifying the Sellmeier model type is considerably more robust to noise. This robustness arises because different gas ratios (as described by Sellmeier models) generate markedly distinct $D_{\text{int}}$ profiles.

**FIGURE 9:**
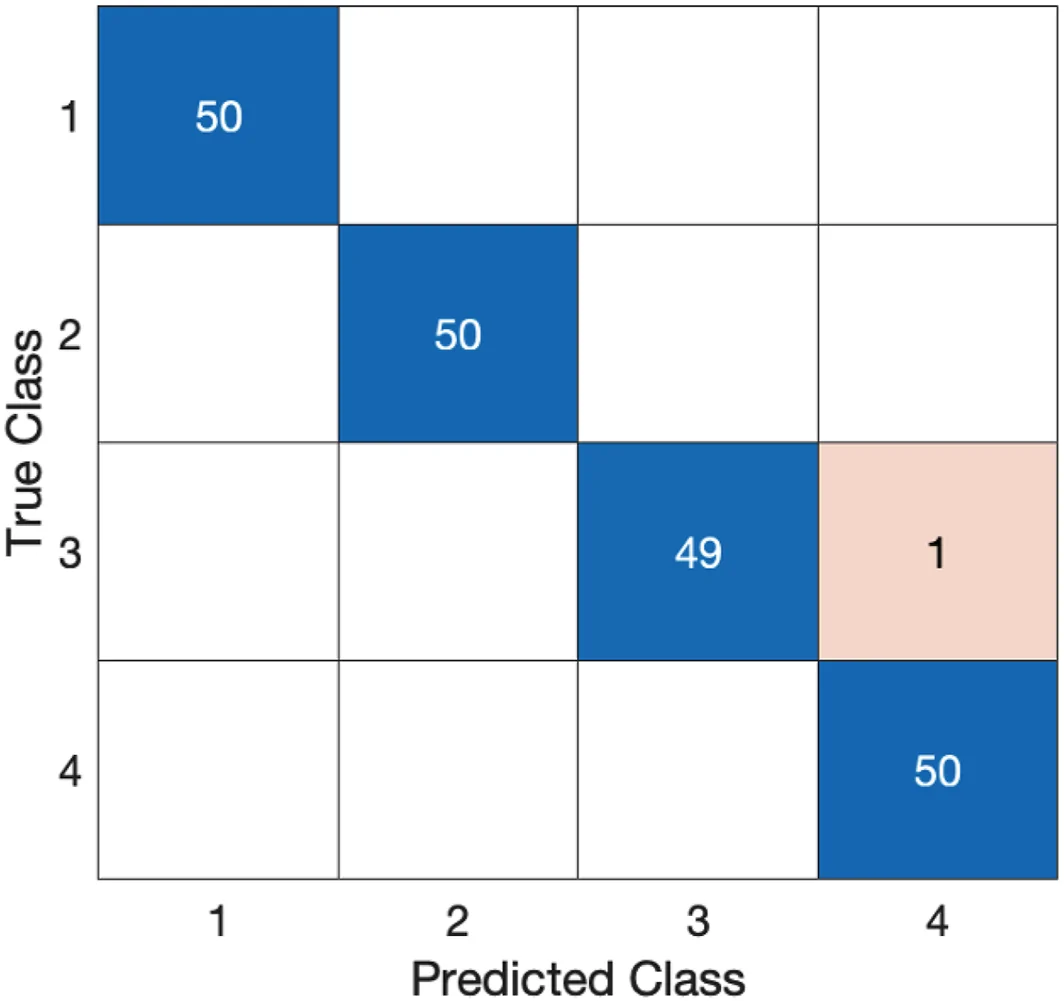
Confusion matrix for the Sellmeier model classification task under all cases (no noise, 50 MHz noise, and 200 MHz noise).

It is worth noting that, beyond classification, one could in principle construct a neural network architecture to directly predict the Sellmeier model coefficients from the measured effective index or integrated dispersion data. However, such a regression task would be significantly more challenging and would require a substantially larger and more diverse training dataset. This is because the effective index and $D_{\text{int}}$ responses are influenced not only by the material dispersion but also by multiple geometric and environmental parameters, including the cladding refractive index, ring radius, width, and height. As a result, disentangling the contribution of the intrinsic material model from these coupled factors would demand an extensive sampling of the parameter space to ensure accurate and generalizable predictions. However, the complexity of this inverse problem could be mitigated by incorporating physical constraints and structured datasets. For example, measurements across multiple nominally identical devices with systematically varied geometries (e.g., ring width sweeps) could be leveraged to decouple material dispersion from geometric contributions, since parameters such as film thickness and refractive index are expected to vary minimally across a local fabrication region. Incorporating such constraints, along with parameter‐sharing or multi‐task learning strategies, may enable more robust extraction of effective material models from dispersion data. Although this approach is promising, it lies beyond the scope of the present work and is an interesting direction for future research.

To determine the minimum number of $D_{\text{int}}$ samples required for accurate classification, we follow a similar strategy as in the previous section: we randomly choose M samples in three regions, yielding a total of N=3M samples. Figure 10 shows the mean classification accuracy as a function of N under three noise conditions: no noise, 50 MHz random noise, and 200 MHz random noise. For small sample sizes (N≤12), the accuracy varies widely, ranging from 80% to 90%, reflecting the limited discriminative information provided by only a few dispersion points. As N increases, the accuracy improves consistently for all cases. With approximately N=21 samples, classification accuracy already exceeds 90%, even in the presence of 200 MHz noise. Beyond this point, the curves rise gradually, with all three noise conditions converging to above 97% accuracy once N≳45. Importantly, the no‐noise and 50 MHz noise cases track each other closely across all N, whereas the 200‐MHz noise case shows slightly lower performance for small N but catches up as the number of samples increases. These results highlight the robustness of the classifier against noise and demonstrate that accurate classification can be achieved with a relatively modest number of $D_{\text{int}}$ measurements (N≈21 to 30), without requiring the complete spectral information.

**FIGURE 10:**
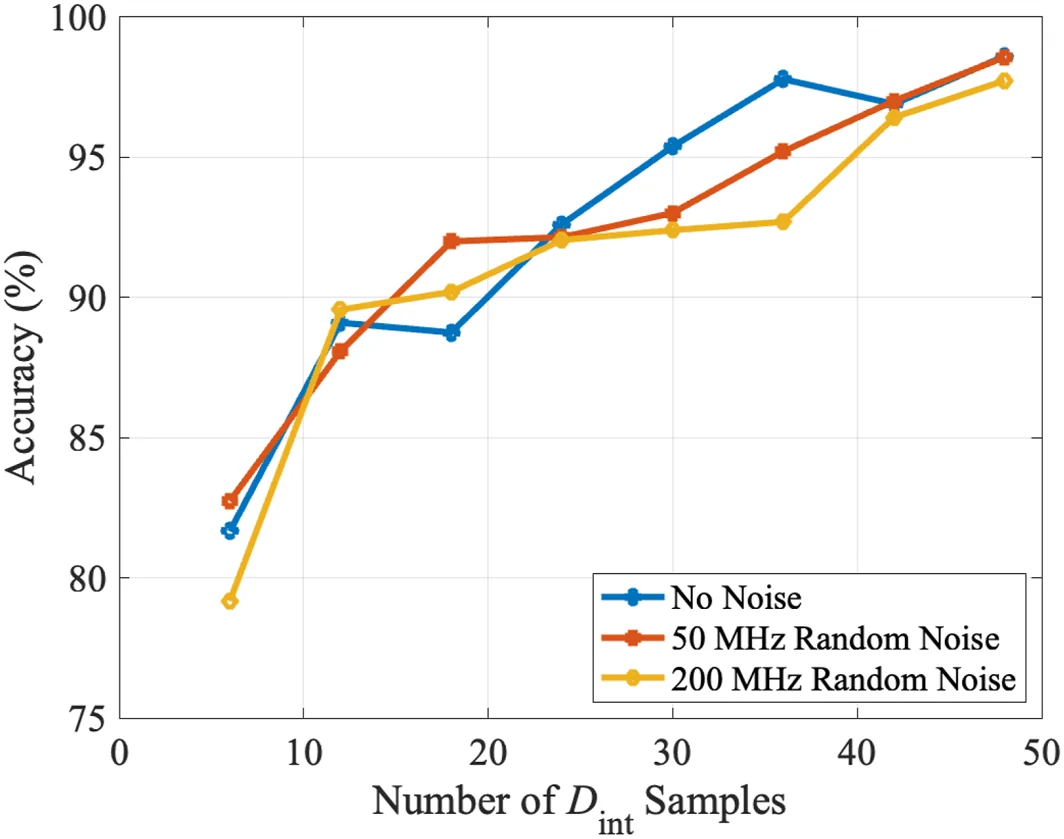
Average Sellmeier model classification accuracy as a function of the total number of $D_{\text{int}}$ samples, N, under no noise, ±50 MHz random noise, and ±200 MHz random noise.

Before we discuss the $D_{\text{int}}$ spectrum prediction, we would like to note that current models are trained on smooth integrated dispersion profiles that do not include localized perturbations, such as avoided‐mode crossings. As a result, the presence of such artifacts in measured spectra can introduce deviations from the learned mapping and may reduce prediction accuracy if they are not properly handled. In practice, however, avoided mode crossings are typically sparse and localized in frequency, and their impact can be mitigated by excluding affected resonances or by selecting $D_{\text{int}}$ samples away from these regions—an approach that is consistent with our strategy of using carefully chosen data points. Alternatively, the framework can be extended by incorporating such features into the training dataset, enabling the network to learn and become robust to these nonidealities. This would require enhancing the dataset with representative perturbations, but it does not pose a fundamental limitation to the proposed approach.

### 3.3 $D_{\text{int}}$ Spectrum Prediction

In our final study, we employ the Sellmeier model for ${\text{Si}}_{3}N_{4}$ from ref. [39] and generate another synthetic dataset following the procedure described in Section 2. The outer ring radius is fixed at 23μm, whereas the ring height and width are varied to construct a dataset for integrated dispersion spectrum prediction. Specifically, the height is swept from 620 to 720 nm in increments of 10 nm, and the width is swept from 800 to 900 nm in increments of 10 nm, resulting in 121 unique training designs.

After building our training dataset, we construct a neural network model, shown in Figure 3c, to predict the integrated dispersion spectrum of a microring resonator directly from the ring width and height. To achieve this, we first normalized the simulated dispersion data and then approximated each spectrum using a sixth‐order polynomial, thereby reducing the high‐dimensional spectral response to a compact representation defined by seven polynomial coefficients. These coefficients, once normalized to ensure numerical stability, served as the output training targets, while the corresponding ring dimensions provided the input features. The neural network architecture was designed with three fully connected layers (64, 128, and 128 nodes), leaky rectified linear unit activations, batch normalization, and moderate dropout rates (0.1, 0.1, and 0.2) to balance model complexity with generalization ability. The training procedure used the Adam optimizer with an adaptive learning rate schedule, early stopping, and $L_{2}$ regularization to mitigate overfitting. Following training, the network was used to infer polynomial coefficients for given test dimensions, and the resulting coefficients were evaluated with a polynomial expansion to reconstruct the full dispersion spectrum.

The red dashed curve in Figure 11 shows the integrated dispersion spectrum calculated for randomly chosen dimensions of RW=867.9nm and RH=664.2nm. When the spectrum prediction network was queried with the same dimensions, we obtained the spectrum shown with a blue solid curve in the same figure. Overall, there is a very good agreement, but we also observe some discrepancies at low μ values (corresponding to high frequencies). To understand the source of this discrepancy, we examined the histograms of the polynomial coefficients used as training data for the neural network and observed that higher‐order terms spanned wider intervals. The distribution of these polynomial coefficients is shown in the (Supporting Information S1: Section S3), illustrating the large dynamic range differences across different orders. This imbalance might have affected the training process, as the neural network may prioritize fitting coefficients with larger dynamic ranges while introducing comparatively higher relative errors in smaller‐magnitude coefficients. Since lower mode indices are more sensitive to the combined contribution of multiple polynomial terms, including those with smaller coefficients, these slight inaccuracies can accumulate and manifest as minor discrepancies in that region.

**FIGURE 11:**
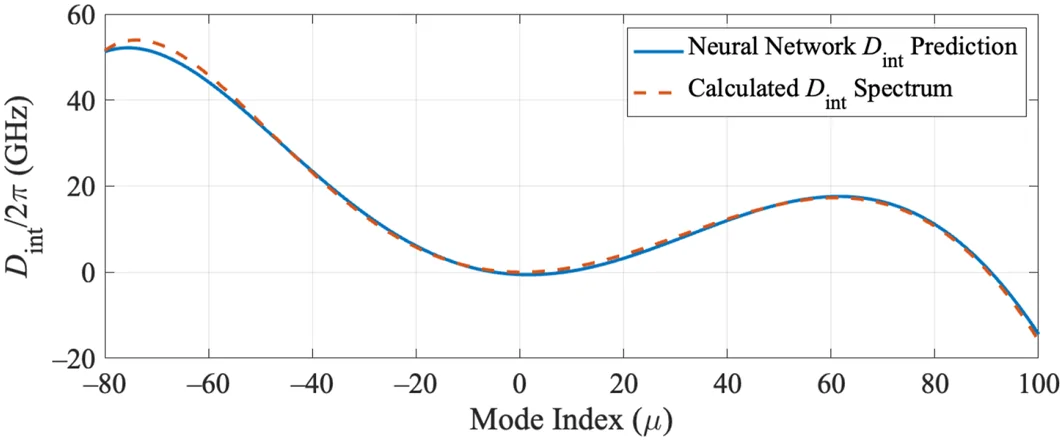
Comparison of dispersion spectra obtained from neural network prediction (blue) and numerical simulation (red dashed curve) for randomly chosen RW and RH values of 867.9nm and 664.2nm, respectively.

Nevertheless, this good agreement and many other successful test cases, which are not shown here, indicate that the network successfully captured the nonlinear mapping between ring geometry and the dispersion profile without introducing spurious oscillations, thereby providing a practical tool for inverse design and accurate dispersion engineering of integrated photonic resonators.

## 4 Experimental Case Studies

### 4.1 FSR of 1 THz

In high‐end photonic foundries, fabrication processes are highly optimized, and a starting assumption of vertical sidewalls (90°) is typically sufficient for first‐order design. However, subtle fabrication tolerances can lead to small deviations from the ideal rectangular profile. To test whether our machine learning framework can resolve these fine structural details, we extended our model to account for a trapezoidal cross‐section, treating the sidewall angle as an additional degree of freedom to improve the fidelity of our dispersion metrology.^1^ We first created a new database using a commercial finite‐element method electromagnetic solver. Here, $w_{b}$, $w_{t}$, and h represent the bottom width, top width, and height of the trapezoids, respectively. The central radius of all rings is 23 μm, and the cladding material (${\text{SiO}}_{2}$) and wavelengths remain the same as in previous sections. The core is silicon nitride, and its refractive index is calculated using the parameters defined in ref. [39].

For the dataset, we generated 105 unique designs by uniformly varying dimensions within the following ranges: $840\;\text{nm}\leq w_{b}\leq 880\;\text{nm}$, $820\;\text{nm}\leq w_{t}\leq w_{b}$, and 660nm≤h≤690nm. Following data generation, we employed five machine learning models to predict the ring dimensions from the integrated dispersion datasets: a sequential neural network (NN), a support vector machine (SVM), extreme gradient boosting (XGB), decision trees (DT), and random forests (RF). Before training, we identified the optimum parameters via hyperparameter tuning. Finally, for testing, we input our measured integrated dispersion data to obtain the predicted dimensions.

Table 6 lists the dimensions recommended by these five machine learning models. We first observe that NN and SVM models predict geometrical parameters within a relatively narrow range.

**TABLE 6:** Dimensions predicted by different machine learning models for microrings with a trapezoidal cross‐section.

ML model	$w_{b}$	$w_{t}$	h
NN	862.29	829.56	672.11
SVM	862.24	831.92	671.93
XGB	859.74	848.75	672.10
DT	870.0	840.0	680.0
RF	863.13	833.93	685.06

We then obtain the effective indices of these rings using the finite element solver and calculate the integrated dispersions. Figure 12 compares the calculated integrated dispersions with the measured ones. The dispersion spectra of the NN, SVM, and XBG designs show the best agreement with the experimental results. At low μ values, there is a significant difference in integrated dispersions of the rings recommended by the DT and RF.

**FIGURE 12:**
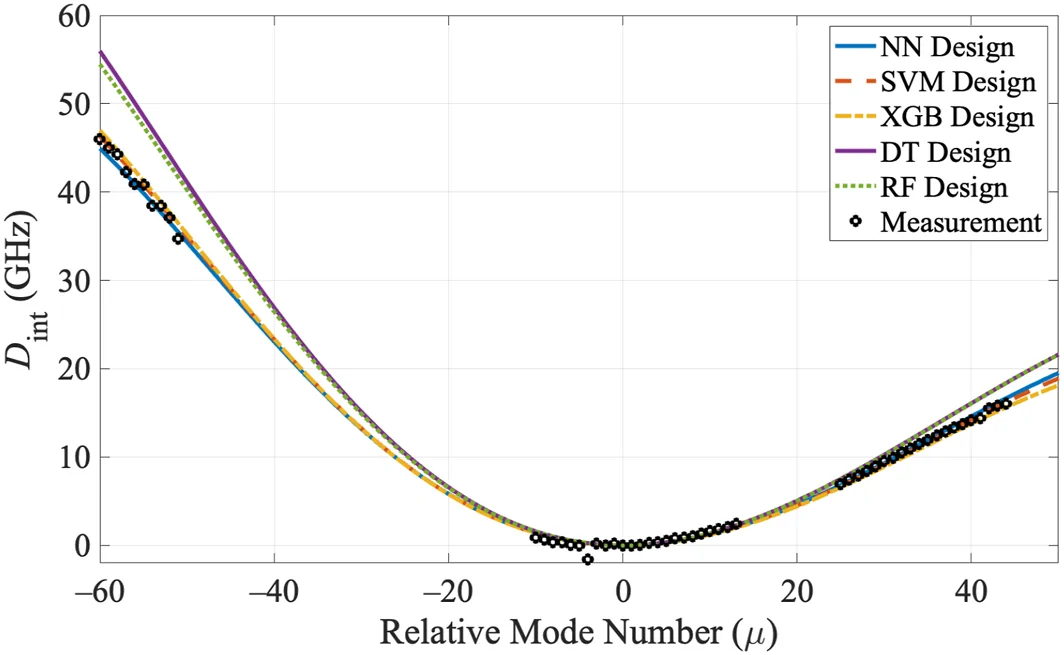
Integrated dispersion spectra of the rings recommended by different machine learning models versus measurement results.

The convergence of the results obtained from the NN and SVM models, as presented in Table 6, highlights a fundamental alignment between their mathematical architectures and the underlying physics of photonic microresonators. In these resonators, optical properties such as the effective index and integrated dispersion evolve as smooth, continuous, and differentiable functions of the device dimensions. The NN and SVM models are particularly adept at capturing this physical continuity due to their inherent ability to perform high‐dimensional interpolation. By employing a tanh activation function in our architecture, the NN enforces a smooth transition across the learned mapping, effectively constructing a continuous manifold that mirrors the wave‐optical behavior of the resonator. Similarly, the SVM utilizes a radial basis function kernel to project the input features into a high‐dimensional space where it can determine a smooth, nonlinear regression surface. Although XGBoost is a tree‐based ensemble, its iterative gradient‐boosting process allows it to refine its predictions through successive corrections of residuals, thereby achieving a level of sub‐wavelength precision that approximates a continuous function. This collective ability to model smoothness explains why these three models yield nearly identical predictions for the ring height (h≈672 nm) and width. In stark contrast, the DT and RF models demonstrate a clear performance gap, characterized by “blocky” or quantized predictions such as the 680.0‐nm height suggested by the DT. This discrepancy is a direct consequence of their reliance on piecewise constant approximations. A DT operates by recursively partitioning the feature space into discrete, orthogonal bins; consequently, it assigns a constant output value to any input falling within a specific leaf node. This “staircase” effect prevents the model from capturing the fine, nanometer‐scale sensitivities required for accurate dispersion engineering. Although the RF attempts to mitigate this by averaging the outputs of multiple trees, it remains fundamentally constrained by the limitations of its base learners. It cannot perform true local interpolation between sparse training points and instead tends toward a local average, which explains the higher bias observed in its predicted dimensions. These architectural limitations make DT and RF less suitable for photonics applications, where even small geometric deviations can cause significant shifts in the zero‐dispersion wavelength.

Based on these results, we infer the height of the resonator to be (672±1) nm and the average width to be ≈850 nm. Notably, the models converge on a sidewall angle θ between 88.5° and 90°. This result is consistent with the foundry's nominal vertical‐sidewall design and illustrates the sensitivity of the ML framework to sub‐degree sidewall deviations. We note that without machine learning methods, the dimensions that best matched the experimental results were 670 and 860 nm, assuming a rectangular cross‐section. Finally, although focused ion beam cross‐sectioning and electron microscopy techniques can, in principle, be used to validate the extracted geometry, the insulating nature of the constituent waveguide materials and the limited imaging contrast between them can limit the ability no better than ±5 nm.

### 4.2 FSR of 100 GHz

Ye et al. [40] fabricated ${\text{Si}}_{3}N_{4}$ ring resonators and reported ring and waveguide height and width at 810 nm and 2 μm, respectively, with their raw data publicly available on Zenodo. The only geometric parameter not explicitly reported was the ring radius; however, given their devices' 100 GHz FSR, one can predict that the radius should be in the 225μm–230μm range.

Using this constraint, we generated a new dataset that spans radius 225μm to 230μm, width 1.7μm to 2.5μm, and height 800nm to 850nm and applied two independent machine‐learning approaches: a feedforward multilayer perceptron (MLP) and a deep kernel learning (DKL) model to infer the ring dimensions from Ye et al.’s measured integrated dispersion data. Both models converged on width and height values (Table 7) in close agreement with the values (2μm and 810 nm, respectively) reported in their paper. Furthermore, the integrated dispersion spectra re‐calculated using these ML‐predicted dimensions show very close agreement with Ye et al.’s measured $D_{int}$ (Figure 13).

**TABLE 7:** Ring radius, width, and height values predicted by two different neural networks.

Network	Radius (μm)	Width (nm)	Height (nm)
MLP	226.9255	2001.4060	802.7887
DKL	225.3002	2018.5762	807.6925

**FIGURE 13:**
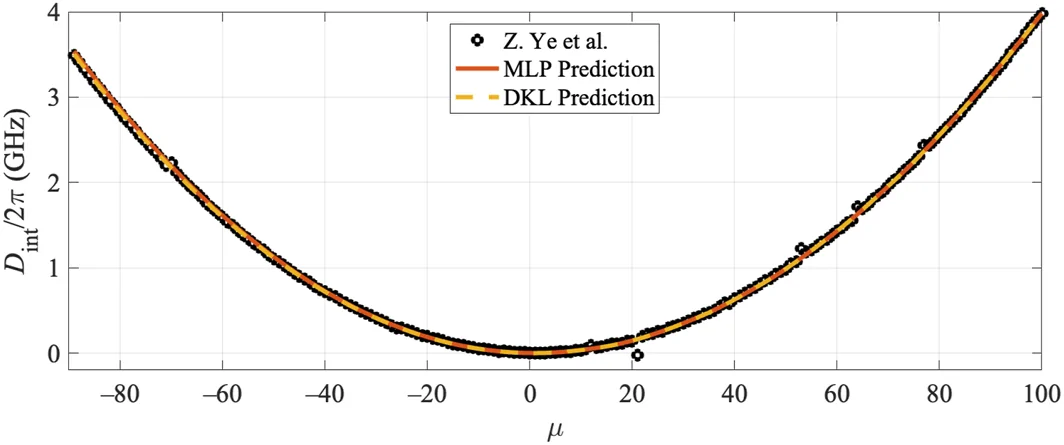
Integrated dispersions measured by Ye et al. and calculated by us using the parameters determined by neural networks.

This independent case study complements the experimental case study of Section 4.1. Here, the ML‐predicted dimensions are compared directly against physically measured values from a separate foundry and device geometry, with the ring radius constrained only loosely by the measured FSR rather than fixed a priori. The close agreement obtained in both width and height, despite this additional radius uncertainty, provides strong support for the accuracy of the proposed frameworks.

Finally, we note that the proposed frameworks are inherently flexible and can be extended to include additional physical and geometrical parameters without altering the methodology. Parameters such as birefringence (e.g., in x‐cut ${\text{LiNbO}}_{3}$) or variations in the cladding refractive index (due to changing environmental conditions or a different material) can be incorporated as additional input features into the neural network. However, increasing the dimensionality of the parameter space generally requires a corresponding expansion of the training dataset to ensure sufficient coverage and to avoid overfitting. In such cases, more advanced sampling strategies (e.g., Latin hypercube sampling) or physics‐informed constraints may be beneficial to efficiently explore the higher‐dimensional design space. Additionally, modest adjustments to the network architecture (e.g., increased width or depth) may be required to capture the more complex relationships between inputs and dispersion response. Overall, although scalability increases data and training requirements, the underlying approach remains directly applicable and well‐suited to multi‐parameter photonic design problems.

## 5 Conclusion

In this work, we demonstrate a nondestructive method for dispersion metrology of foundry‐fabricated devices based on the optical characterization of the integrated dispersion $\left(D_{\text{int}}\left(\mu \right)\right)$ of microring resonators. Importantly, we show that through proper training of a machine‐learning model, a limited sample of judiciously chosen resonance frequencies can enable accurate dimension prediction even in the presence of realistic measurement noise. First, we demonstrated that $D_{\text{int}}\left(\mu \right)$ profiles provide a compact and information‐rich fingerprint for both regression and classification tasks in ${\text{Si}}_{3}N_{4}$ microring resonators. Using numerically generated datasets, we trained neural networks to predict device dimensions and fabrication conditions with high accuracy. For the regression task, the networks achieved sub‐nanometer mean absolute errors in the absence of noise and maintained reliable performance under realistic perturbations, with width predictions being more sensitive to noise than height predictions. For the classification task, identifying the Sellmeier model type associated with precursor gas ratios, accuracies exceeding 99% were obtained and shown to be robust to added noise, reflecting the distinctiveness of the $D_{\text{int}}$ profiles across different gas ratios. Importantly, we found that dispersion samples taken far from the pump frequency are most informative: With a precise measurement system of ±50 MHz deviation, approximately 45 samples are sufficient to predict device dimensions with errors below 8 nm, whereas for less precise systems with ±200 MHz deviation, the expected errors approximately double. Having demonstrated such an approach, we reversed it and also demonstrated the prediction of the $D_{\text{int}}$ profiles directly from ring dimensions, which provides a complementary capability for device modeling and design. Furthermore, two experimental case studies confirm that the proposed framework can handle measured integrated dispersion data. In the first, the framework successfully estimates the sidewall angle of fabricated structures, providing a precision refinement to the nominal vertical‐sidewall assumption and highlighting its potential for monitoring subtle process drifts in an otherwise optimized fabrication flow. In the second, ML‐predicted ring dimensions show close agreement with the reported values from an independently fabricated device, further validating the framework's accuracy across different foundries and device geometries.

Altogether, these findings establish integrated dispersion as a powerful observable for nondestructive and rapid quality control in large‐scale photonic manufacturing, and they offer a generalizable framework for linking measurable optical responses to underlying fabrication parameters, thereby accelerating the development and deployment of advanced photonic systems.

## Author Contributions

**Ergun Simsek:** investigation, writing – original draft, visualization, writing – review and editing, formal analysis, data curation, methodology, validation. **Shao‐Chien Ou:** investigation, writing – review and editing, software, formal analysis, data curation. **Gregory Moille:** conceptualization, writing – review and editing, methodology. **Kartik Srinivasan:** conceptualization, investigation, writing – original draft, methodology, visualization, writing – review and editing, formal analysis, supervision.

## Funding

E.S.’s contribution to this work has been supported in part by cooperative agreements with the National Center for Manufacturing Sciences 2022138‐142232 and 2023200‐142386, which are sub‐contracts from the US‐DoD (http://dx.doi.org/10.13039/100000005) cooperative agreements HQ0034‐20‐2‐0007 and HQ0034‐24‐2‐0001, respectively. S.‐C.O, G.M., and K.S. acknowledge support from the NIST‐on‐a‐chip program and the Space Vehicles Directorate of the Air Force Research Laboratory. K.S. further acknowledges support from the NIST CHIPS Metrology Program and the Microelectronics Commons QUPICS program.

## Ethics Statement

The authors have nothing to report.

## Conflicts of Interest

The authors declare no conflicts of interest.

## Supporting information


Supporting Information S1

## Data Availability

The codes and datasets generated and analyzed in this study are available in the GitHub repository, https://github.com/simsekergun/DeviceMetrology.

## Endnotes

^1^
A detailed comparison between trapezoidal and equivalent rectangular cross‐sections is provided in the Supporting Information (S1: Section S1), where we quantify the resulting differences in $D_{\text{int}}$ and relate them to modal field distributions.
