# Supplementary material for: Microring Resonator Dispersion Metrology With Neural Networks
Source: Nanophotonics. 2026 Aug 31;15(17):e70269. doi: 10.1002/nap2.70269 (PMC13672154; doi:10.1002/nap2.70269)
Supplement: Supplementary file 1 [file NAP2-15-e70269-s001.pdf]

Supplementary Materials

Rectangular vs. Trapezoidal Cross-Section

We consider the following question: how close would the integrated dispersions of ring resonators with trapezoidal and rectangular cross-section be, if the width of the rectangular cross-section is equal to the average width of the trapezoidal cross-section, as illustrated in Fig. 1 (a)?

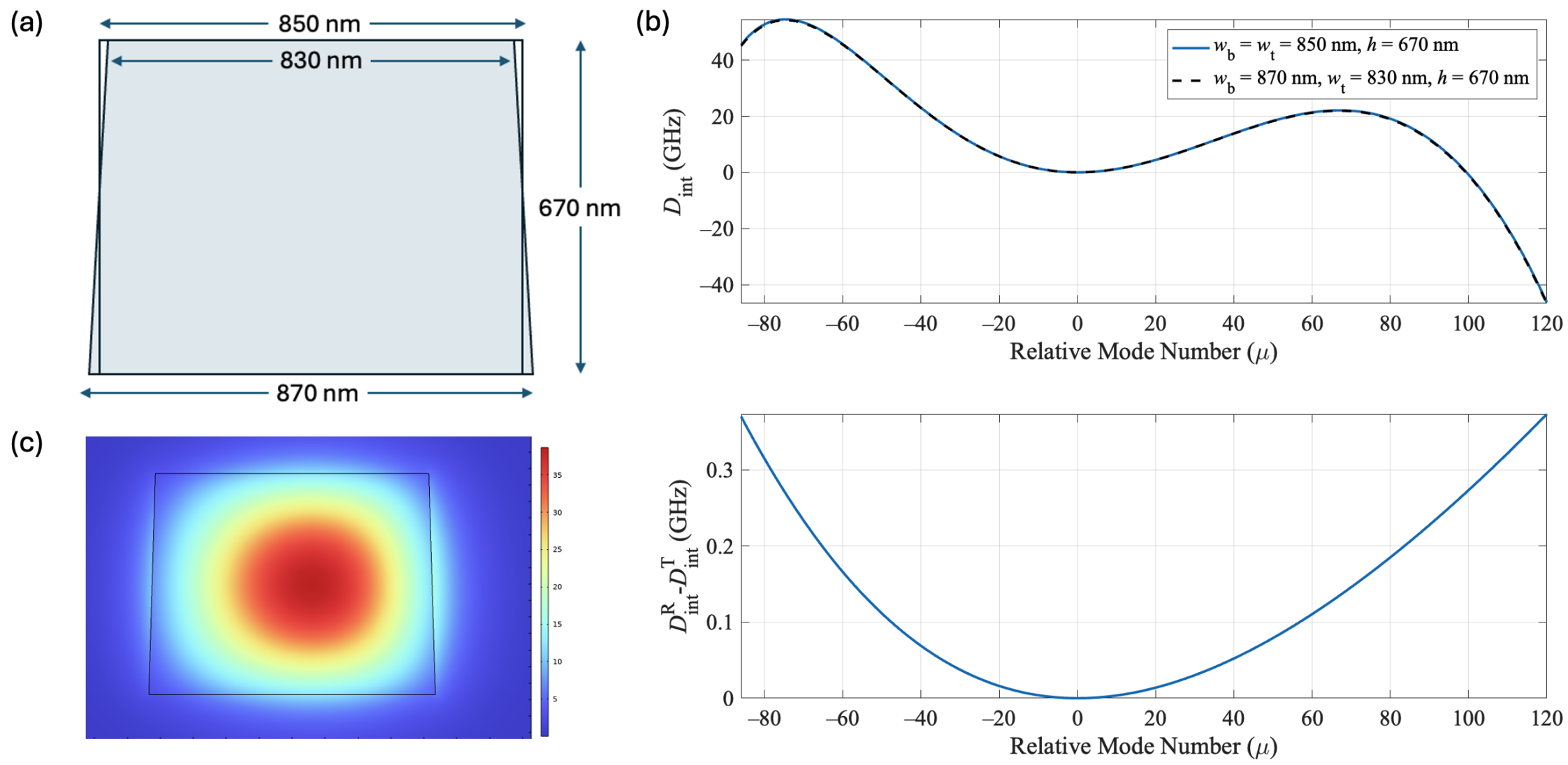

SM Figure 1: (a) A ring resonator with a rectangular cross-section where the width is 850 nm and the height is 670 nm and ring resonator with a trapezoidal cross-section with the same height. Its bottom and top widths are 870 and 830 nm. (b) Top: Integrated dispersions of these Si_3N4 ring resonators. Bottom: The difference between the two integrated dispersion spectra. (c) Electric field intensity around the trapezoidal cross-section.

To answer this question, we calculated the integrated dispersion of two ring resonators. The ring with a rectangular cross-section has a width of 850 nm and a height of 670 nm. The ring with a trapezoidal cross-section has the same height and its bottom and top widths are 870 and 830 nm, respectively, i.e., $w_r = (w_b + w_t)/2$. The upper part of Fig. 1 (b) shows their integrated dispersions and the lower part is the difference between them. The difference is very small and it can be explained with the following two facts: (1) the effective index is determined via a mode overlap integral and (2) for the fundamental mode, the field is strong at the center and weak near the edges as shown in Fig. 1 (c). The difference in D_{int} becomes appreciable (>wavemeter resolution) far away from the pump. This additionally justifies the value of dispersion measurements in multiple, widely separated bands, as we discussed in the manuscript.

Interpolation vs. Neural Networks

In this section, we address the following question: instead of a neural network, can we use interpolation?

To answer this question, we first calculated the integrated dispersion of ring resonators with rectangular cross-sections. The height is 720 nm for all the rings. Ring width (RW) was changed from 820 nm to 890 nm at steps of 5 nm. After removing the RW = 870 nm data from the dataset, we used the "makima" interpolator in MATLAB to predict the integrated dispersion of this RW = 870 nm case. In Fig. 2, the top figure shows the calculated (blue) and interpolated (red) integrated dispersions. The bottom figure is the difference. The difference can reach 3 GHz, which is nearly a 6 % error. Hence, interpolation is not recommended.

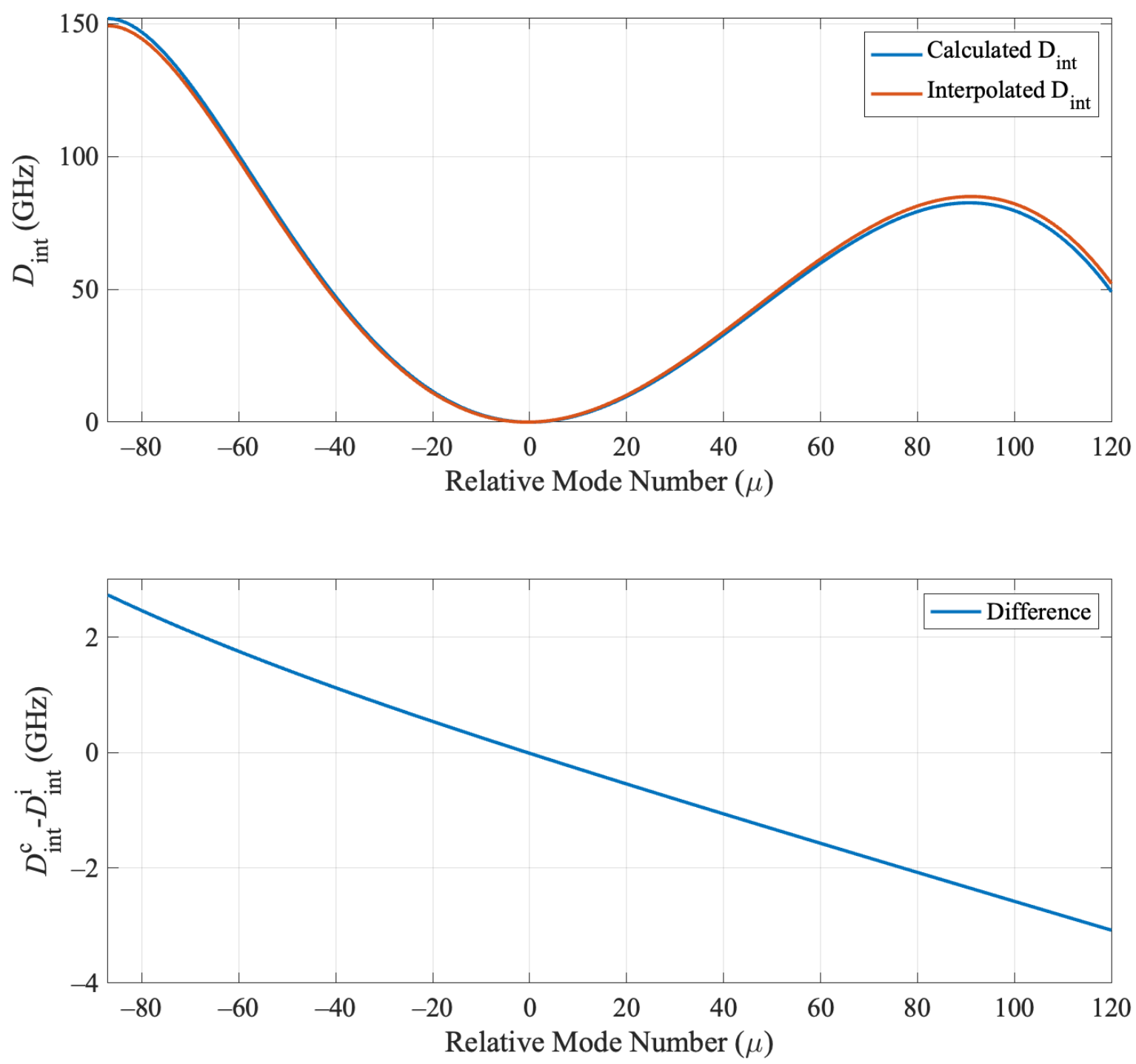

SM Figure 2: Top: Blue curve is the integrated dispersion obtained via a COMSOL Multiphysics simulation for $RW = 870$ nm $RH = 720$ nm. The red curve is obtained via a 2D interpolation from the database, which does not include the results for the same dimensions. Bottom: the difference between these two curves.

Predicting D_{int} Polynomial Fitting Coeffients

Figure 3 shows the histograms of the polynomial coefficients obtained from fitting the integrated dispersion spectra of 545 microring resonators, used as training data for the neural network that predicts dispersion from ring dimensions. We can see that different orders span significantly different numerical ranges, with lower-order coefficients exhibiting relatively small magnitudes and higher-order terms spreading over wider intervals. This imbalance might have affected the training process, as the neural network may prioritize fitting coefficients with larger dynamic ranges while introducing comparatively higher relative errors in smaller-magnitude coefficients. Since lower mode indices are more sensitive to the combined contribution of multiple polynomial terms—including those with smaller coefficients—these slight inaccuracies can accumulate and manifest as minor discrepancies in that region.

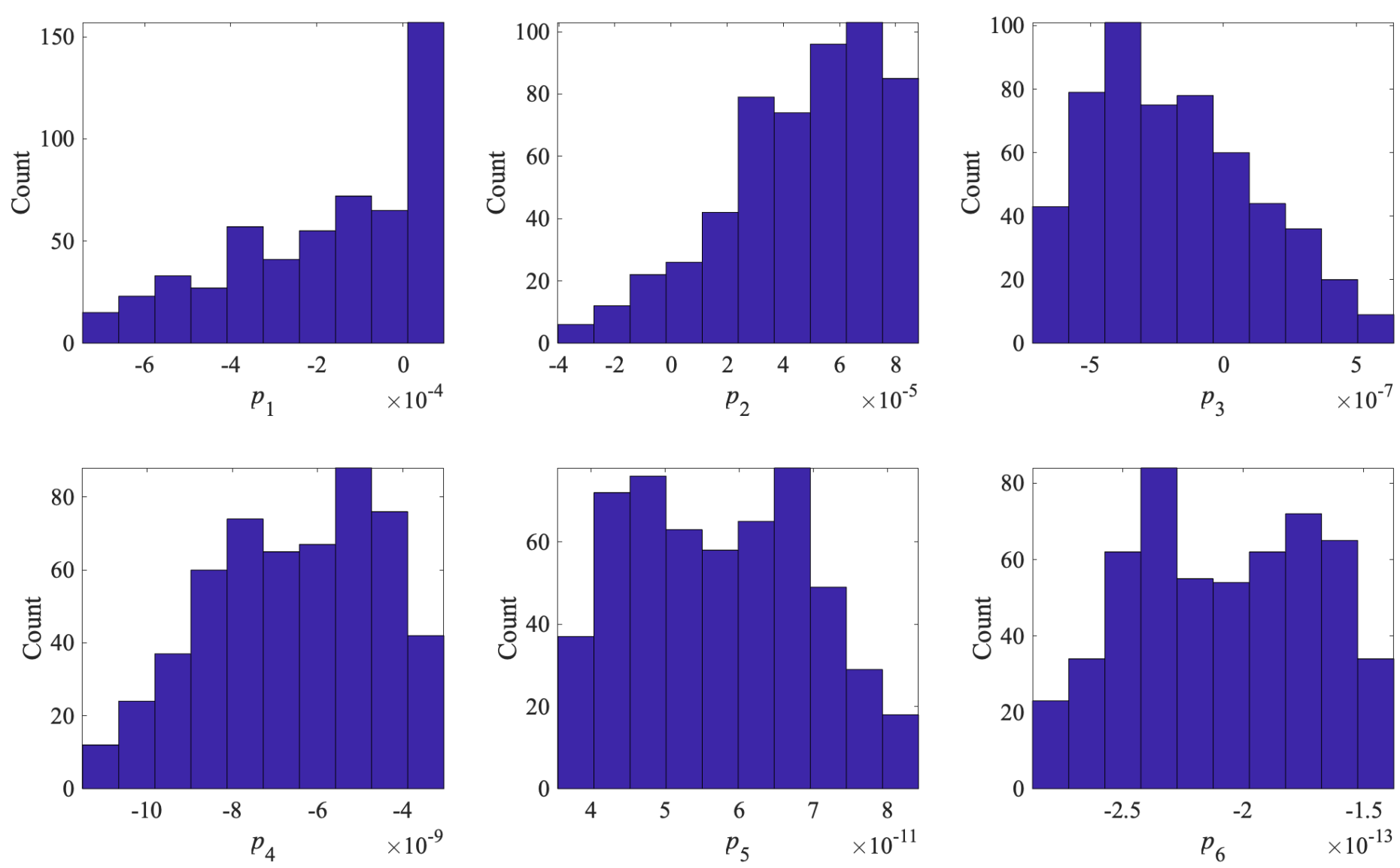

SM Figure 3: Histograms of the polynomial coefficients obtained from fitting the integrated dispersion spectra of 545 microring resonators, used as training data for the neural network that predicts dispersion from ring dimensions.
